# Toward a Global Science of Conservation Genomics: Coldspots in Genomic Resources Highlight a Need for Equitable Collaborations and Capacity Building

**DOI:** 10.1111/mec.17729

**Published:** 2025-03-17

**Authors:** Céline M. Carneiro, Analisa Shields‐Estrada, Alexandra E. Boville, Gabriela Alves‐Ferreira, Tianyi Xu, Ryan L. Wong Arnott, Chloé M. Allen‐Love, Micaela Puertas, John J. Jacisin, Hannah Chapman Tripp, Edmund W. Basham, Kelly R. Zamudio, Anat M. Belasen

**Affiliations:** ^1^ Department of Integrative Biology The University of Texas at Austin Austin Texas USA; ^2^ Programa de Pós‐Graduação Em Ecologia e Conservação da Biodiversidade Universidade Estadual de Santa Cruz Ilhéus Brazil; ^3^ Departamento Académico de Biología Universidad Nacional Agraria la Molina Lima Peru

**Keywords:** adaptive potential, amphibians and reptiles, capacity building, conservation genomics, global climate change, global collaborations

## Abstract

Advances in genomic sequencing have magnified our understanding of ecological and evolutionary mechanisms relevant to biodiversity conservation. As a result, the field of conservation genomics has grown rapidly. Genomic data can be effective in guiding conservation decisions by revealing fine‐scale patterns of genetic diversity and adaptation. Adaptive potential, sometimes referred to as evolutionary potential, is particularly informative for conservation due to its inverse relationship with extinction risk. Yet, global coldspots in genomic resources impede progress toward conservation goals. We undertook a systematic literature review to characterise the global distribution of genomic resources for amphibians and reptiles relative to species richness, IUCN status, and predicted global change. We classify the scope of available genomic resources by their potential applicability to global change. Finally, we examine global patterns of collaborations in genomic studies. Our findings underscore current priorities for expanding genomic resources, especially those aimed at predicting adaptive potential to future environmental change. Our results also highlight the need for improved global collaborations in genomic research, resource sharing, and capacity building in the Global South.

## 1 Introduction

Genetic and genomic resources are powerful tools in biodiversity conservation (Humble et al. 2020; McMahon et al. 2014; Seaborn, Andrews, et al. 2021; Supple and Shapiro 2018). Research shows that combining an understanding of evolutionary and genetic processes with traditional ecological approaches is critical in conservation decision‐making (McMahon et al. 2014; Hohenlohe et al. 2021; Forester, Beever, et al. 2022). Genetic diversity buffers species against global change stressors, from climate change to disease (Savolainen et al. 2013; Bay et al. 2018). Conversely, population declines and extirpations due to global change reduce genetic diversity, which may inhibit responses to future stressors (Bay et al. 2018). Over the last 20 years, genomic resources, defined here as genome‐scale assessments of variation (e.g., whole genomes, or genome‐wide reduced representation datasets) have become increasingly used in conservation and have added power to inferences already afforded by traditional genetic resources such as microsatellites and gene sequences (Forester, Beever, et al. 2022; Heuertz et al. 2023; Theissinger et al. 2023). Genome‐scale data can clarify the capacity of species to respond evolutionarily (through adaptation) to global change stressors, and predicting this response is increasingly important with rapid environmental change (Funk et al. 2019; Seaborn, Griffith, et al. 2021; Kardos et al. 2021; Forester, Beever, et al. 2022; Heuertz et al. 2023; Theissinger et al. 2023). To combat biodiversity loss, many would argue that we need more genomic data from more places across the globe. This raises the question: where are the ***cold spots*** of genomic resources?

The central mechanism by which genomic variation buffers populations against stressors is through increasing adaptive capacity. For this review, we define adaptive capacity as the ability to respond to disturbance, take advantage of new environments, or cope with the consequences of global change. Adaptive capacity confers resilience to new conditions and is therefore tied to extinction risk (Forester, Beever, et al. 2022). The genetic component of adaptive capacity is often referred to as adaptive potential. Here we adopt previous authors' definition of adaptive potential: the ability to evolve genetically based changes in response to selection (Funk et al. 2019; Seaborn, Griffith, et al. 2021; Kardos et al. 2021). Populations with high genomic variation, whether as standing genomic variation or variability in functional genomic responses, have higher adaptive potential and therefore a higher likelihood of persistence (Kardos et al. 2021; Forester, Beever, et al. 2022).

Genetic data are applied to conservation in several ways. Both traditional genetic markers and genome‐scale data can be used to infer historical processes such as gene flow, genetic drift, and selection, which are important for population management (Humble et al. 2023; Meek et al. 2023). Gene flow and genetic drift are often critical for populations that have become small or disconnected from broader metapopulations. Knowing the historical demography of those populations provides a baseline for comparison with populations that are currently responding to stressors (Humble et al. 2023; Frankham 2005; Hohenlohe et al. 2021). Understanding historical gene flow in endangered species is also important for genetic or evolutionary rescue programs (Frankham 2015; Fitzpatrick et al. 2020; Fitzpatrick and Funk 2021). Unlike genetic data, genome‐scale data can inform more directly on the adaptive potential of populations (Meek et al. 2023; Bonnet et al. 2022) and benefit conservation efforts such as captive breeding or reintroductions. For example, data on immunogenomic variation among populations with differing susceptibilities to an emergent disease will inform the best source populations for genetic‐assisted husbandry and population restoration (Kosch et al. 2019). Likewise, understanding the genomic basis of thermal tolerance limits allows us to predict population losses under climate change (Dixon et al. 2015; Tan et al. 2023). As our understanding of the genomic architecture underlying potentially adaptive traits increases, our application of genomics to conservation efforts will broaden (Meek et al. 2023; Hohenlohe et al. 2021).

While genomic data are key to estimating adaptive potential, there are caveats that need to be accounted for when attempting to predict future adaptation (Hoban et al. 2016; Pardo‐Diaz et al. 2015). First, accurate inferences about adaptive variation require adequate sampling and sequencing (Rossetto et al. 2021). Second, for adaptation to occur, standing genomic variation in populations must be responsive to the specific demands imposed by stressors (Feiner et al. 2021) and it is not always possible to know what genomic variation will be important for responding to future stressors (Kardos and Shafer 2018). This is a challenge for conservation genomics as a field because there are many novel stressors that challenge populations alone or in combination. Relationships between genomic processes and effective responses to these stressors are not always straightforward. Third, genomic data are costly to produce and require specialised infrastructure and technologies. Given these caveats, assembling a variety of genetic and genomic resources is an important component of our conservation toolbox (Pardo‐Diaz et al. 2015; Keagy et al. 2023). These resources should include diverse types of data that inform different questions in conservation, including genetic structure and population genetic diversity (e.g., inferred from established neutral markers); reference genomic data (e.g., reference genomes and transcriptomes); genomic variation within and among populations (e.g., spatial distribution of SNPs across a species' range); functional variation associated with global change stressors (e.g., gene expression in cool vs. warm environments); and assessment of adaptive potential (e.g., combining genomic data with forecasting models).

Generating the data needed for effective biodiversity conservation requires global collaboration and coordination. The infrastructure needed to generate genomic data is not equally distributed globally; unsurprisingly, this follows patterns of global socioeconomic disparities (Omotoso et al. 2022; Fitak et al. 2019; Stefanoudis et al. 2021). The most biodiverse and vulnerable regions of the world also have the least resources for producing genomic data for biodiversity conservation, and are concentrated in the Global South (Asase et al. 2022; de Vos and Schwartz 2022; Omotoso et al. 2022; Fitak et al. 2019; Stefanoudis et al. 2021). These global disparities have led to international efforts to increase genome sequencing and make those resources available to the global research community. Examples include the Genome 10K Consortium, established in 2009 with the goal of sequencing 10,000 vertebrate genomes (Genome 10K Community of Scientists 2009), the Global Invertebrate Genomic Alliance (GIGA Community of Scientists et al. 2014), and the Earth BioGenome Project (Lewin et al. 2018), among others (Shaffer et al. 2022; Teeling et al. 2018; Kosch et al. 2024; Robinson et al. 2011; Zhang et al. 2015; Ebenezer et al. 2022). The need for equitable sharing of genomic resources was codified in the Nagoya Protocol, an agreement of the Convention on Biological Diversity. The goal of the Nagoya Protocol is to provide a fair, transparent legal framework for the use of genomic resources, including creating conditions to promote and encourage research contributing to biodiversity conservation and sustainable development (Buck and Hamilton 2011; Kariyawasam and Tsai 2018). While recognition has been steadily growing that leadership by local peoples and inclusive efforts are needed for effective conservation, there remains a need for capacity building and resource sharing, especially in the Global South (Ocampo‐Ariza et al. 2023; Miller et al. 2023; de Vos and Schwartz 2022).

Although many ecosystems are affected by rapid global change, some species are more sensitive than others. Characteristics including organisms' physiological limits, habitat requirements, and interspecific interactions can mediate vulnerability (Foden et al. 2013, 2019). In particular, amphibians and reptiles face significant threats from global change. These taxa typically have low dispersal abilities, strong dependence on climate and specific habitats, and high susceptibility to emerging diseases (Decena et al. 2020; Lorch et al. 2016; Sinervo et al. 2010; Scheele et al. 2019). Although some species exhibit behavioural plasticity in response to global change, there are limits. Rapid increases in temperature can bring ectotherms above their critical thermal maxima, and even less extreme temperature fluctuations can limit foraging and reproduction (Sinervo et al. 2010). Persistence will depend on plasticity of thermal tolerance over shorter time scales (through regulatory genomic processes), and adaptive increases in thermal breadth over longer time scales. In the tropics, where the diversity of amphibians and reptiles is highest, adaptation can be even more challenging: most tropical ectotherms have a narrow thermal safety margin and exist close to their critical thermal maxima (Tewksbury et al. 2008; Polato et al. 2018; Sinervo et al. 2010). Thus, even small increases in temperature could compromise fitness for a large number of species. For these reasons, studies of adaptive potential are particularly important for amphibian and reptile conservation.

Here, we perform a systematic review of peer‐reviewed literature to assess the global availability of genomic resources for amphibians and reptiles. First, to evaluate geographic biases in genomic resources and how these align with conservation needs, we georeference genomic resources and overlay these onto distribution maps of amphibian and reptile species diversity and predicted climate change. Second, we assess the scope of published genomic resources based on their potential applicability to global change. Specifically, we classify genomic resources into five scope categories: (1) General genomic resources; (2) Spatial genomic variation; (3) Functional genomic variation related to global change; (4) Functional genomic variation related to climate change specifically; and (5) Adaptive potential or future vulnerability to climate change. These five categories represent genomic datasets that range from reference genomes (scope level 1) to assessment of adaptive potential in response to a specific global stressor (scope level 5). We examine the distribution of these five categories of genomic resources globally and compare them to IUCN vulnerability categories for each species. Third, we evaluate geographic patterns in author institutions to assess collaborations in genomic studies, how authorship patterns relate to the genomic scope of studies, and the prospects for local capacity building. We provide our results as a resource to facilitate prioritisation of genomic resource development and local research capacity in the most at‐risk and understudied regions.

## 2 Methods

### 2.1 Literature Search and Data Filtering

We conducted a literature search in January 2024 using Web of Science (Core Collection), Agricola, and SciELO. We compiled a keyword list of terms related to genetics and genomics, amphibians and reptiles, adaptation, evolution or variation, and climate and environment (Table S1).

We deduplicated papers resulting from our search and imported them into Rayyan (Ouzzani et al. 2016) for two rounds of collaborative filtering (Figure S1). In the first round, we included papers if: (1) focal species were amphibians and/or reptiles and (2) the study generated genomic‐level data (Table S2; Figure S2). If only one of two initially assigned reviewers included a paper, a third reviewer was randomly assigned for tie‐breaking. In the second round of filtering, three reviewers were assigned to each paper to confirm that the study generated new genomic data rather than using data from public databases.

### 2.2 Data Extraction

A three‐person subset of coauthors determined the type and scope of genomic data. Scope was divided into five categories representing potential applicability to global change: Level 1—general resource (e.g., reference genome or transcriptome); Level 2—spatial variation (e.g., ddRAD data from multiple populations); Level 3—functional variation relevant to global change but not specifically climate change (e.g., differential gene expression in response to chemical pollutant); Level 4—functional variation specifically relevant to climate change (e.g., differential gene expression in response to altered temperature); and Level 5—adaptive potential (e.g., genomics‐informed forecasting models). If multiple genomic data types and/or scope levels were included in a paper, these were recorded as distinct genomic resources, and thus as separate entries in our dataset (See Table S3 for additional details on genomic data types). We then randomly assigned coauthors to all papers to extract data on species identity, geographic locality, and author affiliations (Table S3). We grouped each study according to global region (Global North vs. Global South) using the UN Conference on Trade and Development's classification of economies (UNCTAD 2023). To avoid taxonomic discordance and disagreements about species delimitations, we updated genera according to IUCN (2024) and otherwise retained taxonomy as originally published by the authors.

### 2.3 Data Analysis

To identify coldspots, global areas lacking in genomic data relative to species richness, we obtained per‐country amphibian and reptile species data from public databases (Uetz et al. 2024; Frost 2024) and divided the number of species with genomic resources by the species richness in each country. We then assessed the distribution of genomic resources relative to climate change risk. We obtained mean annual maximum temperature (*T*_max_) for the periods 1961–1999 (historical) and 2041–2060 (future) from the Worldclim 2.1 database at a resolution of 2.5 arc min (Fick and Hijmans 2017). For future climate projections, we used SSP585 (Shared Socio Pathways—SSP585) compiled by the Coupled Model Intercomparison Project (CMIP6), using three General Atmosphere–Ocean Circulation Models—AOGCMs: MIROC6, IPSL‐CM6A‐LR, and MPI‐ESM 1‐2‐HR (Cannon 2020). SSP585 is considered a pessimistic scenario, which assumes that CO2 emissions will triple by 2075 and the increase in global temperature will be between 3.3°C and 5.7°C by 2100, and is typically used to reflect “worst case scenario” under continued high emission regimes. We then calculated the magnitude of expected change at each pixel (ΔT_max_ = future maximum temperature minus historical maximum temperature). We extracted the ΔT_max_ values for each point in our dataset using the package terra (Hijmans et al. 2022).

Second, we explored how genomic resources of different scopes are distributed globally. We visualised genomic scopes of each study (Levels 1–5) across continents (North America, South America, Oceania, Europe, Asia, Africa) (Wickham 2016). We evaluated the relationship between scope level and continents or study type (captive vs. wild animals) using chi‐squared tests. We examined the distribution of genomic resources across species by conservation status category, which we retrieved for each species from the IUCN database (IUCN 2024). Species were classified into eight IUCN categories: Data Deficient (DD), Least Concern (LC), Near Threatened (NT), Vulnerable (VU), Endangered (EN), Critically Endangered (CR), Extinct in the Wild (EW) and Extinct (EX). We deduplicated our data such that each species was represented once per unique combination of study × scope level × genomic data type and then evaluated the distribution of scope levels across IUCN categories using a chi‐squared test.

Third, we examined geographic patterns in authorship using a network connecting the country of each first author's institutional affiliation to the country of field sampling in each study (wild animal studies only) using the R packages *ggraph* (Pedersen 2024) and igraph (Csárdi et al. 2023). We extracted degree centrality values using *igraph* to examine differences between papers first‐authored by researchers affiliated with institutions in the Global North vs. Global South. We used a Wilcoxon Rank Sum Test to evaluate the relationship between sampling region and number of local authors, defined as authors with institutional affiliations in the country where sampling took place.

## 3 Results

### 3.1 Dataset

Our literature search resulted in 9919 studies (Carneiro et al. 2025). After screening titles and abstracts, we retrieved full text for 1707 studies to assess eligibility for second round screening and data extraction (Figure S1). Our final dataset consisted of 693 published genomic studies of amphibians and reptiles across 145 countries (Table 1a), including 610 studies that included wild species and 98 studies that included captive species (Table S4). Among the 693 unique studies, 330 included amphibians and 365 included reptiles (two studies included both taxa). In total, our dataset included 1382 species (557 amphibians and 825 reptiles; Table 1a). More than half of the studies (57.72%) used reduced‐representation sequencing (GBS/RAD/sequence capture) approaches; over one‐third of studies (35.64%) used functional genomic approaches (transcriptomics, traditional RNASeq, and RNA capture arrays); and the remaining studies reported whole‐genome sequence data (10.97%; Table S5); genomic approaches were relatively evenly distributed among amphibian and reptile species (Table S6). In total, we recovered 2658 unique genomic resources, defined as all unique combinations of study identity, genomic data type, genomic scope level, country sampled, and species identity.

**TABLE 1:** Summary of data in this study. (a) Total number of studies, species, and countries by taxonomic group. (b) Mean percentage of species richness with genomic resources calculated by country and averaged for the Global North, Global South, and all countries included in our dataset by taxonomic group. Note that overall, our dataset includes 6.6% of all amphibian and reptile species described globally (1382/20,914). (c) Genomic resources in five scope categories (ranked according to their applicability to conservation under global change) and their distribution across the studies of amphibians and reptiles (*n* = 693). Numbers in the Total column are sometimes not the sum of the amphibian and reptile columns because some studies report on species from both taxonomic groups, report on multiple species, or include sampling from multiple countries.

(a) Count	Amphibia	Reptilia	Total
Study	330	365	693
Species	557	825	1382
Country	115	108	145

(b) % Species with Genomic Resources	Amphibia	Reptilia	Combined
Global North	30.76%	10.45%	20.22%
Global South	15.99%	2.65%	7.78%
All Countries	21.13%	4.84%	11.37%

(c) Scope	Amphibia	Reptilia	Total
Level 1: General genomic resource	122	130	252
Level 2: Spatial genomic variation	144	178	320
Level 3: Functional variation–global change	41	23	64
Level 4: Functional variation–climate change	18	33	51
Level 5: Adaptive potential	10	6	16

### 3.2 Distribution of Genomic Resources Across Species and Climate Change Risk

**FIGURE 1:**
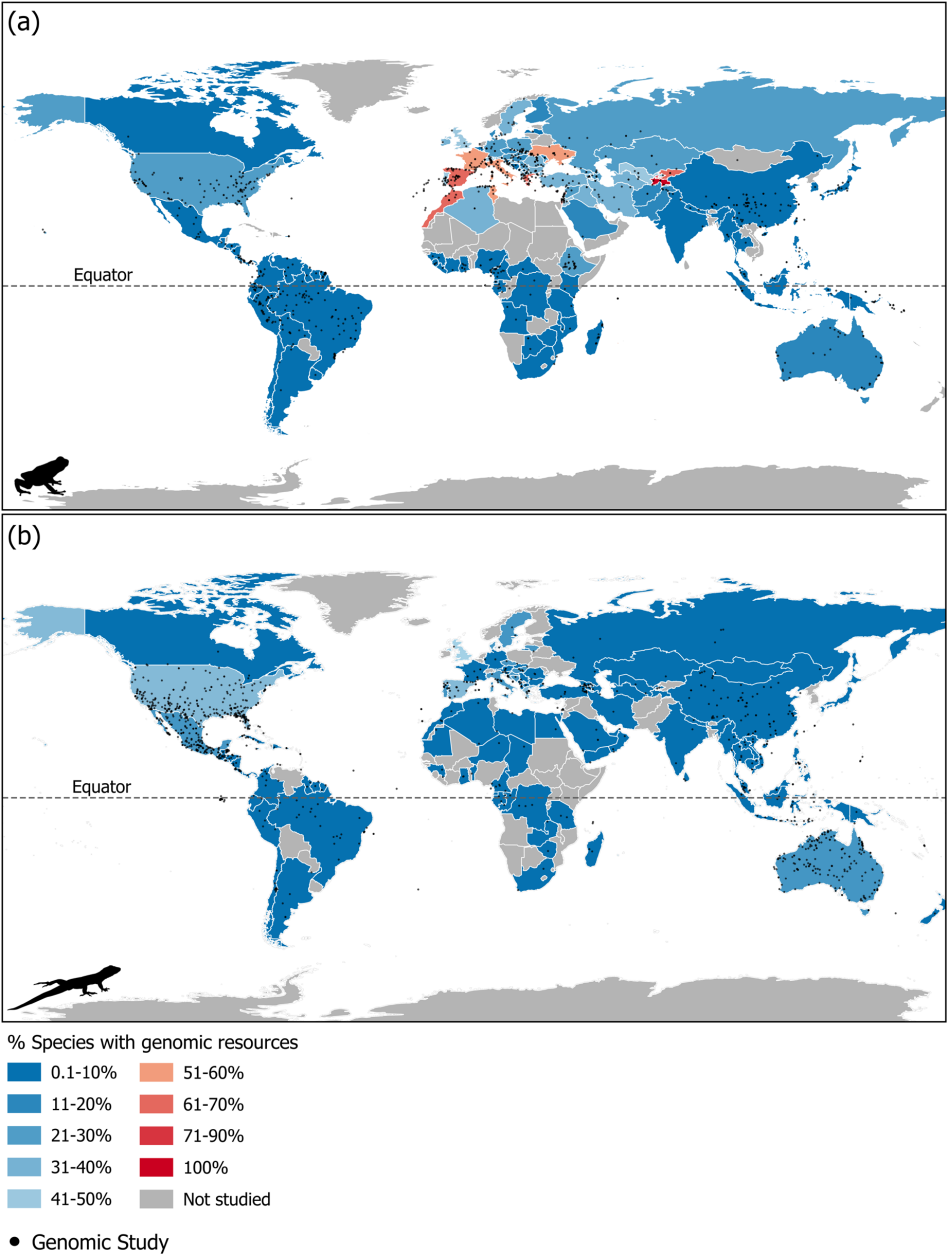
Proportion of amphibian (a) and reptile (b) species in each country with published genomic resources recorded in our dataset. Proportions were calculated as the number of species represented in genomic studies divided by species richness for each country. Grey points represent geographical locations of studies.

Genomic resources for wild species were concentrated in the Global North (Figure 1), with 390 studies covering 625 species. In contrast, the Global South had 285 studies covering 751 species (Table S7). Across continents, most studies involved sampling in a small subset of countries, including the United States (*n* = 199 studies), China (*n* = 80), Australia (*n* = 65), Mexico (*n* = 48), Spain (*n* = 37), Brazil (*n* = 32), France (*n* = 32), and Italy (*n* = 27) (Supplemental File 1). North America had the highest number of studies (*n* = 331), while Africa had the lowest (*n* = 34).

**FIGURE 2:**
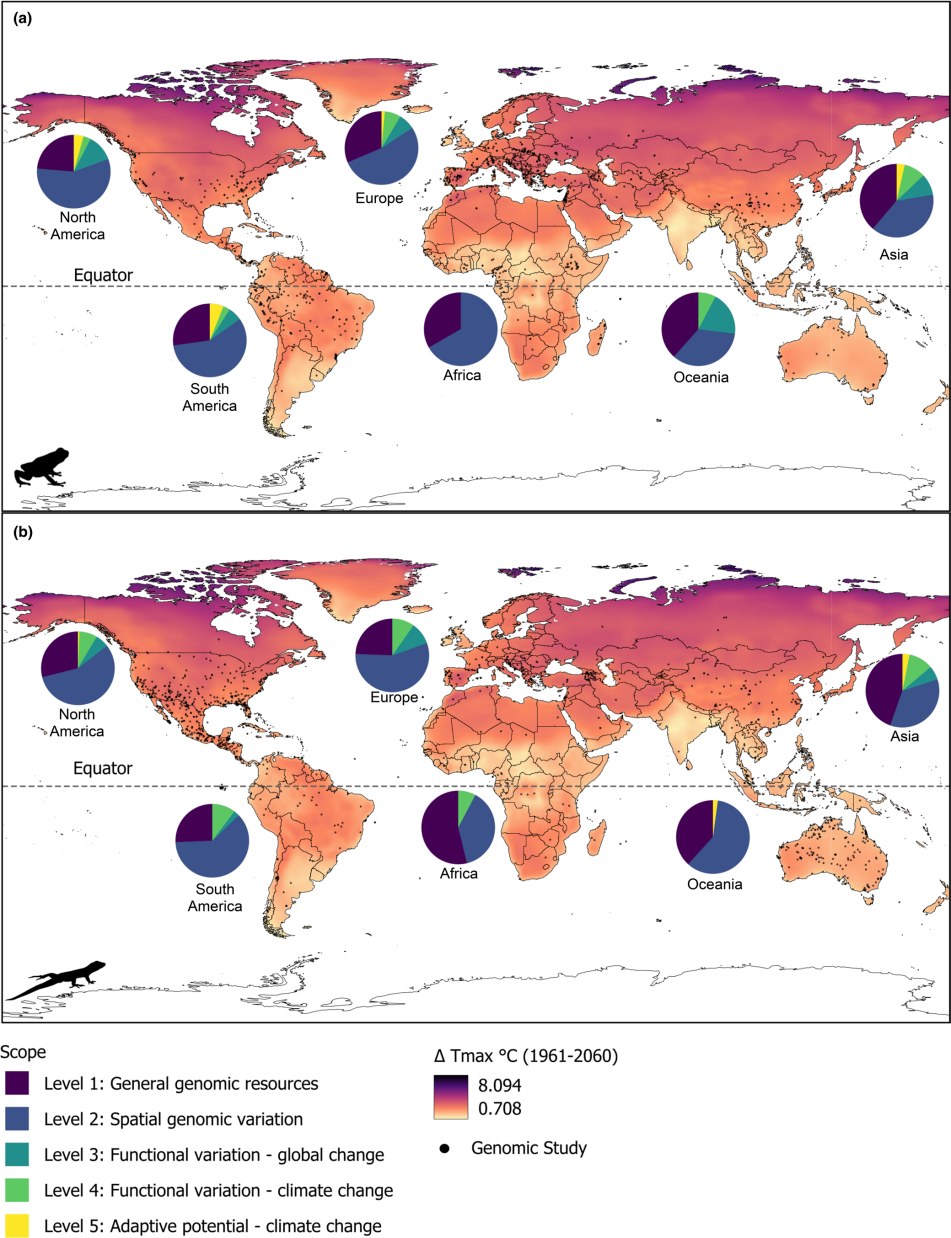
Global distribution of published genomic resources for (a) amphibians and (b) reptiles relative to the maximum predicted change in global temperatures from 1961 to 2060. Pie charts show the proportion of studies from each continent across genomic scopes. Increasing scope level indicates increasing applicability to conservation in the face of global change; see text for additional details. Grey points represent approximate geographic locations of studies.

We estimated the proportion and number of species present in each country for which genomic resources were available. Overall, we recovered genomic resources for 6.6% of amphibian and reptile species worldwide; an average of 11% of species from countries included in our database had genomic resources (Table 1). The percentage of species for which there are published genomic resources in the Global North was over double that for the Global South (20% vs. 8%; Table 1b; Table S7). This is particularly stark when considering total species richness per country; as expected, Global South countries have higher species richness but a lower percent of species with genomic resources (Figure S3). Only 13 countries, primarily located in Europe, Northern Africa, and Central Asia, had genomic resources for ≥ 50% of their amphibian species (Figure 1a). However, even in Global North countries with high amphibian diversity, genomic resources were not abundant. For example, the United States and Australia had genomic resources for 26% (87/338) and 13% (33/253) of local amphibian species, respectively (Figure 1a). Biodiversity hotspots in the Global South showed even lower proportions of genomic resources for local amphibian species. Notably, less than 1% of amphibian species had published genomic resources in India (0.7%, 3/431), Honduras (0.7%, 1/152), and Thailand (0.5%, 1/208; Figures 1a and 2a). Africa had the largest number of countries with no published amphibian genomic resources (27/54), followed by Asia (22/47) and Europe (19/50; Figure 1a).

We observed a similar pattern for reptiles, but with a lower percentage of species with genomic resources compared to amphibians (5% vs. 21%, respectively; Figure 1b; Table 1b; Figure S3). Four countries from the Global North had genomic resources for more than 20% of reptile species: United States (31%, 171/558), United Kingdom (40%, 2/5), Greece, and Spain (both 21%, 16/77). Among Global South countries, Mexico had the highest percentage of reptile species with genomic studies (11.59%, 118/1018) followed by China (7.76%, 50/644); however, many biodiversity hotspots in the Global South had very low percentages of species with genomic studies, including Brazil (2%, 20/878), Thailand (1%, 5/501), South Africa (0.4%, 2/570), and India (0.3%, 2/806). Our search recovered no genomic resources for reptiles from more than half of the countries in Oceania (10/14), Africa (33/54), and Europe (27/50) (Figure 1b). Across the globe, the most drastic ΔT_max_ occurred in northern high‐latitude regions; predicted increases in ΔT_max_ declined toward the equator. Because genomic studies were concentrated at higher latitudes, these largely overlap with areas of greatest ΔT_max_ (Figure 2).

### 3.3 Scope of Genomic Resources and Their Relevance for Conservation

Clustering of genomic studies at higher latitudes (20°–50° N) held true across all genomic scope levels (Figure S4). General genomic resources (Level 1; 36%) and spatial genomic variation (Level 2; 46%) comprised the majority of genomic resources in our dataset, while fewer reported functional variation related to global change (Level 3; 9%), climate change (Level 4; 7%), and adaptive potential (Level 5; 2%; Table 1c; Table S8; Figure S5). Proportionally fewer genomic studies in Levels 3–5 were reported for reptiles compared to amphibians, despite the higher number of reptile studies in the dataset. We recovered a significant difference in the research scope of genomic studies using captive versus wild organisms (χ^2^ = 2863.4, df = 10, *p* < 0.0001; Table S4). Most captive studies focused on general genomic resources, while approximately half of the wild studies investigated spatial genomic variation. Additionally, a larger proportion of captive studies evaluated functional genomic variation related to global change relative to wild studies.

General genomic resources and spatial variation studies were evenly spread across levels of climate change vulnerability. Studies including higher‐level genomic resources (Levels 3–5, addressing functional variation and adaptive potential) were fewer but generally included sampling of wild populations in areas with higher climate change vulnerability (Figure 2; Figure S6).

In North America, South America, and Europe, most genomic data focused on spatial genomic variation (Level 2), followed by general genomic resources (Level 1; Figure 2). In addition, South America had a large percentage of studies exploring adaptive potential (Level 5) for amphibians, but not for reptiles. Asia had roughly equal numbers of studies on general genomic resources and spatial genomic variation, with a consistent proportion addressing higher‐level genomic resources. Oceania primarily focused on general genomic resources and spatial genomic variation for amphibians, and spatial genomic variation for reptiles. African studies primarily provided general genomic data, with few exploring functional variation in both groups and spatial genomic variation in reptiles.

**FIGURE 3:**
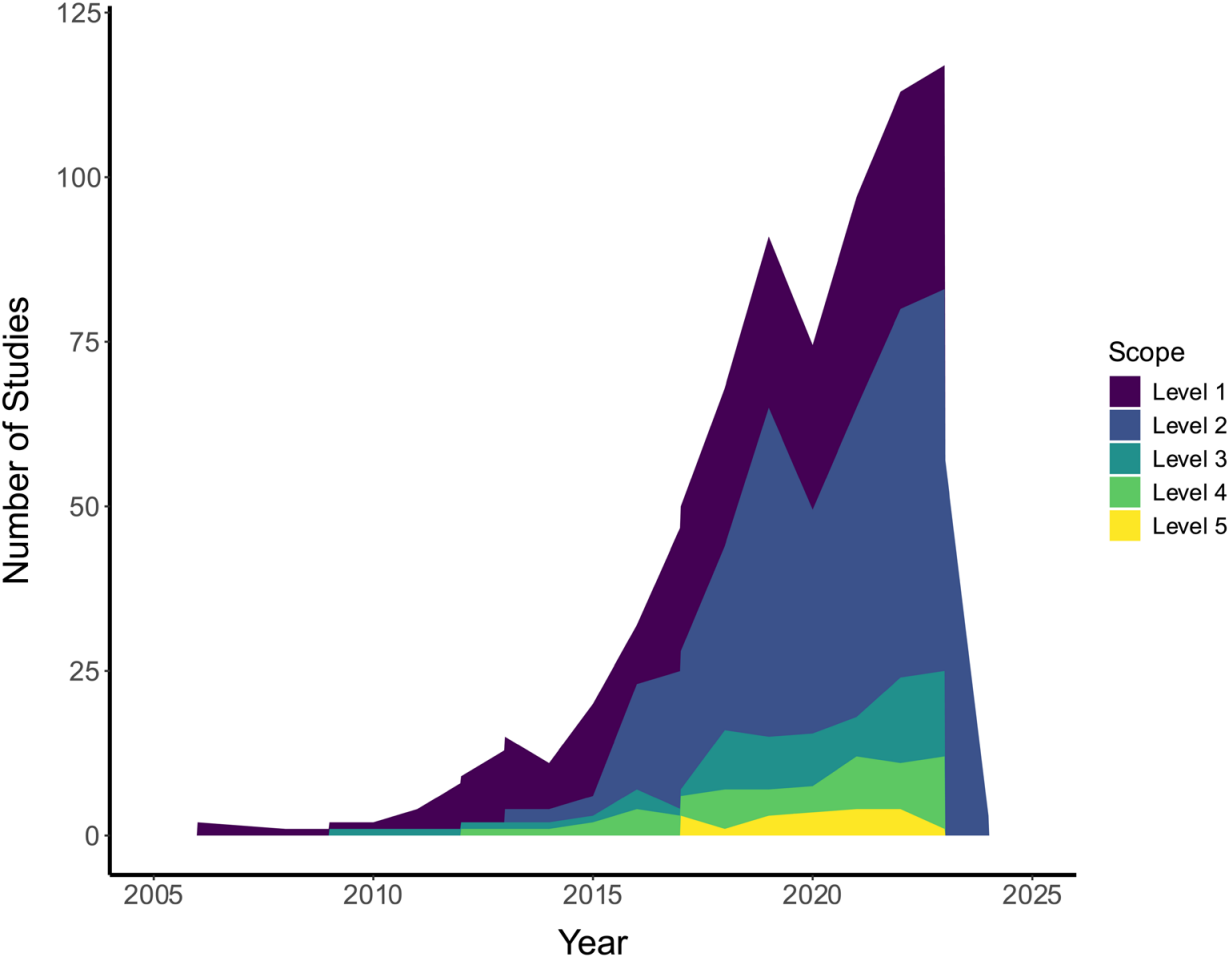
Number of studies of captive and wild animals with genomic resources in each of the genomic scope categories across publication years 2005–2024 (*n* = 693 unique studies; 10 studies included multiple scope levels). Increasing scope level indicates increasing applicability to conservation in the face of global change; see text for additional details.

Genomic research on amphibians and reptiles has increased over time, most drastically from 2014 to 2024 (Figure 3). Level 1–2 studies (general genomic resources and spatial variation) increased the most, while Level 3–4 studies (functional variation) grew more moderately through time. The first Level 5 studies (adaptive potential) were published in 2017, with fewer than five published each year from 2017 to 2023.

**FIGURE 4:**
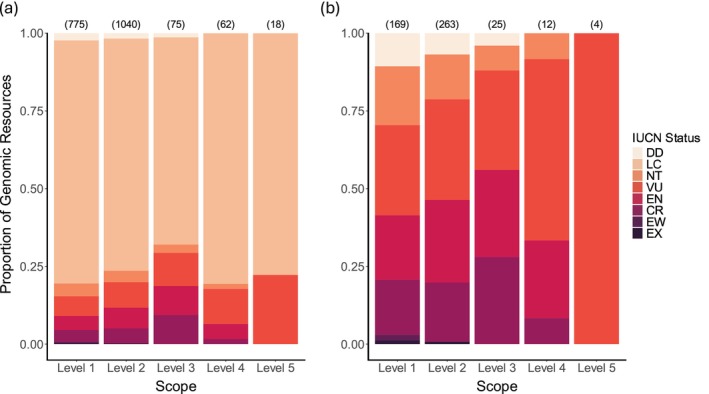
Proportion of published genomic resources for wild and captive species across IUCN threat categories (DD = data deficient, LC = least concern, NT = near threatened, VU = vulnerable, EN = endangered, CR = critically endangered, EW = extinct in the wild, EX = extinct) and genomic scope levels. Increasing scope level indicates increasing applicability to conservation in the face of global change; see text for additional details. A genomic resource is defined as a unique combination of study identity, scope, and genomic data type for a particular species. (a) Proportion of genomic resources for species of all threat categories (*n* studies = 672; *n* genomic resources = 1933). (b) Proportion of genomic resources for species of all threat categories except LC (*n* studies = 235; *n* genomic resources = 436). Numbers above bars represent the total number of genomic resources in each scope level.

IUCN status was available for 1,244/1,382 species in our dataset (Table S9). The majority of the species were Least Concern, with few species in higher threat categories (Table S9). This pattern was evident across data types and scope levels (Figure S7; Figure 4a). While there was no significant association between scope and IUCN status (χ^2^ = 30.777, df = 24, *p* = 0.1603), Level 3 studies (functional variation related to global change) included relatively more Near Threatened, Vulnerable, Endangered, and Critically Endangered species. Level 1 studies (general genomic resources) included relatively more Endangered species than other levels (Figure 4b).

### 3.4 Patterns in International Collaborations

**FIGURE 5:**
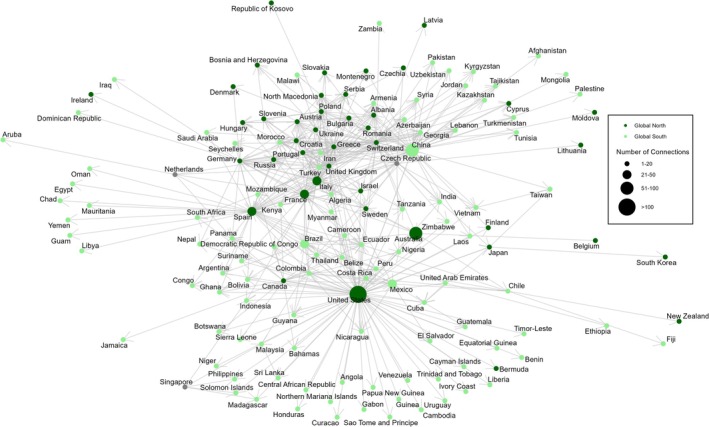
Global collaborative author network for all published genomic resources included in our review. The network reflects patterns of international work by first authors at institutions in countries of the Global North (dark green) and Global South (light green). The size of each country node represents the number of connections with other countries (international sampling locations), and the arrows represent the direction of the connection, going from the country where the first author is affiliated to the country where the focal species were collected.

We observed a clear asymmetry in authorship of genomic studies. Papers led by authors with affiliations in the Global North have significantly more sampling (field efforts) in the Global South than vice versa (Figure 5; Figure S8). Authors from Global North countries mostly occupy central positions within the authorship network with many international connections across the globe, indicating high numbers of international studies and a prominent role in the conservation genomic research landscape. In contrast, most Global South countries are peripheral, indicating fewer collaborative connections and reduced integration into the global amphibian and reptile genomics research community (Figure 5). These differences are reflected by a reduced degree of centrality in studies with first authors affiliated with institutions in the Global South relative to those led by Global North authors (Global South mean degree centrality = 2.45, ± 0.163 SE; Global North = 8.38 ± 1.24; Figure S9). Exceptions include China, Mexico, and Brazil, which are more integrated in the collaboration network compared to other Global South countries.

**FIGURE 6:**
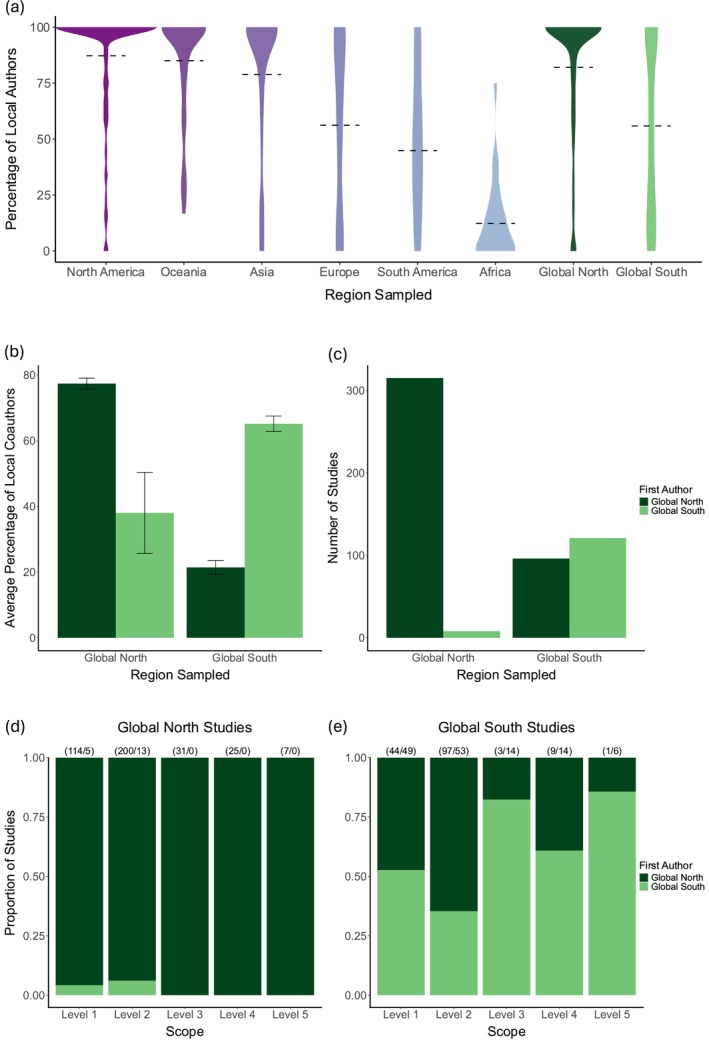
Trends in authorship by region sampled and location of institutional affiliation of authors for studies of wild animals with sampling locality data. (a) Percentage of local authors by region sampled. Percentage of local authors is defined as authors with a primary institutional affiliation in at least one of the countries where the study's sampling occurred. (b) Percentage of local coauthors (not including the first author) averaged across studies, grouped by global region sampled and global region of the first authors' primary institutional affiliations. (c) Total number of studies by global region sampled and the global region of the first authors' primary institutional affiliations. (d, e) Proportion of studies first‐authored by researchers with institutional affiliations in the Global North or Global South, involving sampling in the Global North (d) or Global South (e), across genomic scope levels. Increasing scope level indicates increasing applicability to conservation in the face of global change; see text for additional details on genomic scope. Numbers above bars indicate the number of studies first‐authored by researchers from the Global North followed by the number of studies first‐authored by researchers from the Global South. For aggregation by continent in (a), 53 studies were excluded because they included sampling in multiple continents (n studies included = 556). For aggregation by global region in (a–c), 54 studies were excluded because they included sampling in both global regions (*n* studies included in a = 555); in (b, c) 4 single‐author studies were also excluded (*n* studies included in b, c = 551). In (d, e) the 54 studies having sampling in both global regions are included in both panels (*n* studies = 609, *n* genomic resources = 673); genomic resources, defined as all unique combinations of study identity, scope level, and global region.

Of the 610 studies focusing on wild amphibians and reptiles, only three were single‐authored; in all three studies, the authors were local (i.e., had institutional affiliations in the country where field sampling occurred). Among papers with two or more co‐authors, 303 studies were conducted by domestic co‐author teams and 304 by international teams, with similar breakdowns for both amphibian and reptile studies (Table S10; Figure S10). Most genomic studies were led by first authors affiliated with institutions in North America (283) and Europe (139). Only one study was first‐authored by a researcher affiliated with an institution in Africa. In co‐authored studies involving sampling in North America, Oceania, and Asia, 79%–87% of authors had local affiliations. This percentage dropped to 45%–56% local authorship in studies of wild animals in Europe and South America, and 12% local authorship in studies of wild animals in Africa. In studies involving sampling in the Global North, 82% of authors were local to the country where sampling took place. In contrast, studies involving sampling in the Global South had 56% local authors (Figure 6a).

Overall, lead authorship patterns varied between the Global North and Global South. Approximately 78% of first authors were affiliated with Global North institutions, whereas 22% were from Global South institutions. Last author differences were even more pronounced, with 82% of last authors from the Global North and 18% from Global South institutions (Tables S10 and S11).

When we examined collaboration patterns by study location, we found that the proportion of local coauthors depended on whether the first author was affiliated with a Global North or Global South institution (Figure 6b,c). In studies that exclusively sampled in the Global North, those with a first author from the Global North had 77% local coauthors, while those with a first author from the Global South had 38% local coauthors. The vast majority of studies in the Global North were first‐authored by researchers from the Global North (98%). Among studies that exclusively sampled in the Global South, those with first authors from the Global North had 21% local coauthors, whereas those with first authors from the Global South included 65% local coauthors. First authors of studies that sampled in the Global South were relatively evenly split among regions (44% Global South first authors vs. 56% Global North).

In studies conducted in the Global North, those with first authors from the Global South included only Levels 1–2 (general genomic resources and spatial variation; Figure 6d). Conversely, studies conducted in the Global South with higher genomic scope levels had proportionally more first authors from the Global South (≥ 60%; Figure 6e).

## 4 Discussion

### 4.1 Global Patterns of Genomic Resource Coldspots for Amphibian and Reptile Conservation

Our systematic review examined patterns in genomic resources for amphibian and reptile conservation and patterns of global collaboration. A surprising finding was that genomic resources for amphibians and reptiles were very low on a global scale. We recovered published genomic resources for only 6.6% (1382/20,914) of named amphibian and reptile species worldwide (Uetz et al. 2024; Frost 2024). We also observed significant global biases in the availability of genomic resources, especially when compared with species richness, predicted climate change, and species of conservation concern. Our results indicate large regional cold spots for genomic resources. Not surprisingly, genomic resources were concentrated in the Global North; this coincides with areas of the globe with higher predicted climate risk (the temperate zone, 30°–50° N). Most studies published general genomic or spatial resources, and very few directly estimated adaptive potential. The majority of studies were led by first authors affiliated with institutions in the Global North. Although genomic studies are often the product of international collaborations, we identified a clear asymmetry in local authorship, with studies conducted on species in the Global South having fewer local co‐authors, particularly when these were led by first authors from the Global North. This suggests a need for expanding local capacity building and equitable collaboration between Global North and Global South countries. We discuss each of these major results and the implications of our findings for future efforts in conservation genomics.

### 4.2 Genomic Resource Coldspots Are Concentrated in the Global South

We recovered fewer genomic studies and a smaller proportion of native species with published genomic resources from the Global South; this corroborates findings from a recent survey of published reference genomes across all tetrapods (Linck and Cadena 2024). Many Global South countries are exceptionally species‐rich, making them coldspots for genomic resources. For example, India and numerous countries in South America are in the top 10 countries for amphibian diversity yet have genomic resources for less than 10% of native species. For reptiles, the five countries with the highest species richness are Australia, Mexico, India, Brazil, and Indonesia, and of these, only Australia and Mexico have genomic resources for at least 10% of native species. These patterns indicate that other socio‐economic and political factors, and not conservation needs, contribute to biases in the distribution of genomic resources. These disparities are most evident in some countries in Africa, South Asia, and Southeast Asia for which we recovered zero genomic resources. These countries warrant careful attention, as they host high numbers of endemic species and are often highly vulnerable to global change (Powers and Jetz 2019).

Climate predictions show increases in monthly maximum temperatures globally, with the greatest increases at higher latitudes; this coincides with more published genomic resources for amphibians and reptiles in northern regions. This seems promising, but a few caveats make this an overly optimistic view of ectotherm conservation. First, while these genomic resources are valuable for increasing understanding of adaptive potential, these highly productive regions for genomic research have relatively low species diversity, and thus represent a small fraction of species globally. Second, species in genomic coldspots at lower latitudes are still experiencing significant environmental change that will continue to cause detrimental effects. As ectotherms, amphibians and reptiles are uniquely sensitive to environmental conditions and can experience major shifts in physiology, reproductive biology, and habitat suitability with minor changes in ambient temperatures (Mi et al. 2023; López‐Alcaide and Macip‐Ríos 2011). Thus, we should not underestimate the consequences of climate change on ectotherm biodiversity, despite lower predicted temperature increases in the tropics.

Three countries from the Global South appear to be emerging leaders in genomic studies of herpetofauna. China had the second highest number of genomic studies overall, second only to the United States. Researchers at Chinese institutions produced a substantial number of genomic studies on amphibians and reptiles, likely due to recent economic growth allowing for increased resource allocation for biodiversity research (Zhang et al. 2017; Wang et al. 2020; Wei et al. 2021). Nonetheless, the proportion of species in China for which there are genomic resources is low (0.1%–10%; Wei et al. 2022; Fan et al. 2020). The other two emergent leaders in the Global South were Mexico and Brazil, which ranked fourth and sixth, respectively, for the number of genomic studies. Both countries have experienced rapid deforestation and other threats to biodiversity, but both countries have also recently seen high federal investment in biodiversity research (UNESCO 2010). Both Mexico and Brazil are megadiverse countries, so despite the high rankings in genomic research, these resources are capturing a low percentage of species richness (Figure S3). China, Mexico, and Brazil also had the highest number of collaborative authorship connections of all Global South countries (Figure 5). These countries are clearly leaders in genomic research and could serve as synergistic forces in capacity building in Global South conservation genomic networks.

### 4.3 Do Available Genomic Resources Align With Conservation Needs?

Genomic methods are more accessible than ever before and provide exceptional opportunities to generate critical resources for conservation. In particular, reduced‐representation approaches (GBS/RAD/sequence capture) remain the most accessible methods for non‐model species; indeed, these dominate our dataset. Yet, different types of genomic data inform on different mechanisms that contribute to species persistence (Forester, Beever, et al. 2022; Funk et al. 2019; Nicotra et al. 2015). This raises the question: are we collecting the types of genomic data that maximise our needs for conservation?

Our study revealed notable patterns in genomic resources as they relate to applicability to global change. The number of genomic resources is growing; however, resources related to functional variation and adaptive potential (Level 3–5 scope) are still relatively rare. We recovered only 16 studies explicitly testing for evidence of adaptive potential (Level 5). The majority of these studies focused on amphibians (70%), and most involved Least Concern species (78%). The most common approaches used in these studies included genomically informed common garden or transplant experiments and differential gene expression across treatment groups, while a few paired genomics with other measures of plasticity (e.g., phenotypic) to assess adaptation. All of these approaches require significant infrastructure and funding, but as new methods emerge, experiments may not always be necessary for robust inferences of adaptive potential. A few Level 5 studies used models to assess adaptive potential; these have been successfully applied in other taxa (Bay et al. 2018; Forester et al. 2023) and represent a promising avenue for researchers without access to laboratory facilities. Although these studies can require higher investment in data production, many have argued that understanding local adaptation has enormous benefits for conservation (Meek et al. 2023; Hohenlohe et al. 2021). Studies on adaptive potential will likely continue to increase over time, especially as novel methods for extracting information from genomic data emerge (Wold et al. 2021).

For purposes of this review, we ranked genomic resources into five scope levels based on potential applicability to global change research. However, we recognise that different genetic and genomic resources provide value in many conservation contexts and expect that the conservation genomic toolbox will continue to expand. Forester, Beever, et al. (2022) recommend using multiple “proxies” or frames of reference, to better estimate adaptive potential, combining baseline genomic data and phenotypic and/or environmental data. Data on spatial genomic variation among populations (Level 2) supplement studies on functional or adaptive diversity (Level 3–5) by providing estimates of population‐level genome‐wide diversity. In other words, Level 2 studies provide the foundation for future Level 3–5 studies, which is promising given the abundance of Level 2 studies worldwide. Our review showed that scope level does not increase in areas of high predicted climate change, nor does it increase for taxa with higher IUCN threat categories. This is consistent with previous calls for expanding local adaptation studies in at‐risk taxa (Meek et al. 2023) and underscores that the strategic development of genomic resources is important for further integrating genomics and conservation action.

Further integrating studies of adaptation in conservation biology will not only benefit target species but will also address broader questions about evolutionary mechanisms underlying organismal responses to new challenges (Bonnet et al. 2022). Specifically, do organismal responses draw on a limited number of mechanisms, or does every organism have its own idiosyncratic response? The question of whether adaptation follows similar pathways in different organisms, either through the reuse of genes or gene pathways, is important in conservation because it helps us predict the generality of responses across taxa. Studies of parallel adaptation in plants to alpine environments show that closely related species exhibit parallel signatures of selection on genes involved in adaptation to cold temperatures, short seasons, and increased radiation, but parallel adaptation decreases when comparing more distantly related taxa (Bohutínská et al. 2021). In the context of conservation, genetic divergence between populations or species may affect the pool of shared potential adaptive alleles, and therefore will impact our ability to predict responses with common genomic markers. Future studies on the genetic basis of adaptive phenotypes in threatened taxa may help us develop predictions about the likelihood of evolution vs. evolutionary constraints at the genomic level.

While we advocate for the use of genomic data in conservation, we also acknowledge that much data already exist that are suitable for evaluating historical demography and genome‐wide diversity (a proxy for adaptive potential) that are not genomic (Mittell et al. 2015). These data are often more accessible financially and can sufficiently serve as robust markers for informing conservation practices. However, the benefits of adding genomes to conservation efforts are not incremental. The high density of markers from genome‐scale data provides more accurate estimates of both genetic diversity and greater power to examine evolutionary processes from non‐model species with complex demographic histories (Shafer et al. 2015; Supple and Shapiro 2018; Theissinger et al. 2023), particularly in species with large genomes such as amphibians.

### 4.4 Authorship, Equitable Science, and Capacity Building in Conservation Genomics

The geographic network of authors vs. sampling locations showed that first authors affiliated with institutions in the Global North occupied central “hub” positions (Figure 5) indicating that they performed more international work. Across all studies, 80% had lead authors, and 82% had senior authors affiliated with institutions in the Global North. We found substantial differences in the participation of local authors across regions, best exemplified by the difference between studies conducted in North America (87% of studies had local authors on average) and those conducted in Africa (12% local authors). In addition, we found that very few studies that sampled taxa in the Global North were led by first authors with institutional affiliations in the Global South, while studies that took place in the Global South were split relatively more evenly in first author institution (44% vs. 56% Global North vs. Global South first author). Notably, studies with sampling in the Global South and first authors from the Global North included on average only 21% local coauthors. Taken together, these patterns are consistent with parachute science, a well‐documented phenomenon in which scientists from Global North countries extract data and knowledge from Global South countries without meaningfully collaborating with or providing benefits to local communities (Stefanoudis et al. 2021; Mwampamba et al. 2022; de Vos and Schwartz 2022). Regardless of the cause of the observed authorship patterns, it is clear that work is needed to engage and support researchers from the Global South in conservation genomics.

Our results confirm known biases; the Global South remains under‐resourced and under‐funded in STEM, including in biodiversity research (Miller et al. 2023; Soares et al. 2023). Biological research outside of wealthy universities is a path with many obstacles, including access to equipment, technologies, infrastructure, and funding. Moreover, while species do not recognise geopolitical boundaries, funding agencies and country‐level institutions do. This poses different challenges to Global South researchers in forming collaborations, which may also contribute to the relatively lower number of international studies stemming from Global South researchers in our authorship network (Figure 5). The solution to these biases involves more than just provisioning materials and equipment to Global South institutions. Global South scientists need to be supported through culturally competent local capacity building, respected as leaders, and deferred to as experts on local ecosystems and conservation needs (Barber et al. 2014; Johnson et al. 2022; Utset 2024; Haelewaters et al. 2021).

While the majority of genomic studies overall were led by researchers with institutional affiliations in the Global North, our data show that proportionally, studies with Global South first authors had higher relevance to conservation under global change (Levels 3–5). Previous work shows that when Global South authors lead studies, they have a higher number of local researchers on their teams, and that following the lead of local researchers and communities improves conservation research and outcomes (Price et al. 2023; Keppel et al. 2012). It is critical that we work to build intentional collaborations at all steps of the research process, from project design and fieldwork to authorship in international peer‐reviewed journals, to elevating Global South researchers to leadership roles in professional societies and journal editorial teams (Campos‐Arceiz et al. 2018; Ramírez‐Castañeda et al. 2022). This will in turn enhance the portfolios and reputations of all collaborators, build expertise, and translate into more research opportunities, grants, and positive conservation impact. Adopting best practices in data sharing, data sovereignty, and international collaborations will enhance the field of conservation genomics (Armenteras 2021; de Grijs 2015). These practices will go a long way toward rebuilding lost trust and overcoming the legacy of decades of parachute science (Barber et al. 2014).

### 4.5 Caveats and Potential Areas for Improvement

Our geographic analyses are dependent on geopolitical data, and we recognise that historical precedents resulting from colonialism affect the interpretation of our results. For example, our methods categorise countries as members of the Global North and the Global South according to UNCTAD (2023). This database is inherently biased; for example, several nations across the Caribbean, Africa, and South America (e.g., Puerto Rico, The Canary Islands, and French Guiana) are considered part of the Global North due to the designation of their “parent” country. However, these regions more closely align with the Global South, as they are socioeconomically under‐resourced and are centres of high biodiversity and conservation concern.

We used first and last author institutional affiliations as a proxy for author geographic location and in turn a measure of participation/inclusion of authors across global regions. This may misrepresent authorship, especially if researchers from under‐resourced groups are working from labs in Global North countries. In addition, some authors have multiple affiliations, which in many cases include institutions in multiple global regions. We considered only the first institutional affiliation in our authorship analyses to avoid assumptions that might bias our findings. While this is a best practice for systematic reviews (Partelow et al. 2020), this may impact our results if the order in which affiliations are listed is non‐random. Global South researchers may choose or be invited to spend time at Global North institutions to access resources, whether as students or visiting scholars. In addition, some senior Chinese authors have formal affiliations at Global North Institutions (typically in the US or Europe), but their primary affiliation is a Chinese institution. These cases affect whether a study is classified as a domestic or international collaboration. Despite these potential misclassifications, the observed international coauthorship patterns strongly confirm previous studies and perspectives in Ecology and Evolution (Stefanoudis et al. 2021; Miller et al. 2023) and underscore the need for equitable and inclusive practices in research collaborations (Armenteras 2021).

We note that the division of authors into Global North and Global South is a coarse classification and does not account for intersectional identities of authors that can play a role in research and collaborations (Crenshaw 1991; Tseng et al. 2020). We chose to avoid assigning authors to nationalities or identity groups based on name or institutional affiliation. Using limited publicly available information to assign identity is problematic as it involves assumptions and can lead to misidentification or oversimplification of identities. We also acknowledge that regardless of region, researchers experience unequal access or receive unequal credit for their contributions to research. A number of papers have pointed out the need for improved representation of women and gender minority herpetology researchers in the Global South (Meneses et al. 2024; Cyriac et al. 2022). Field safety and equitable authorship practices remain significant concerns for marginalised scientists worldwide (Demery and Pipkin 2021). The opportunity remains to improve equity and inclusion in genomics research even in countries that appear to be more resourced and productive.

Finally, we considered a single measure of global change, ΔT_max_; this metric cannot capture the true diversity of factors that threaten biodiversity. We classified studies according to one of five genomic scopes (Levels 1–5), of which one specifically considered genomic resources addressing global changes not directly related to climate (Level 3). A number of studies in this category included genomic resources relevant to organismal responses to disease, pollution, and land use change, all of which are present threats to many terrestrial vertebrates (Powers and Jetz 2019; Scheele et al. 2019; Bernanke and Köhler 2009). Likewise, climate variables other than temperature are expected to change as well, including seasonality, precipitation, and the frequency of extreme events (Konapala et al. 2020; Orlowsky and Seneviratne 2012). Future studies should consider the utility and availability of genomic resources that inform about other global change stressors.

### 4.6 Toward a Global Science of Conservation Genomics

The integration of genetic, and later genomic, data in conservation planning and management has an interesting history. The field of conservation genetics became prominent in the early 1980's (Schonewald‐Cox et al. 1983; Falk and Holsinger 1991; Ellstrand and Elam 1993). Even earlier, conservation biologists realised that theoretical inferences from population genetics are directly relevant to metrics including effective population size, inbreeding, and population fitness (Shaffer 1981; Frankham 2005; Frankel 1974). At the time, application of such data was limited by the types and scale of genetic information available for threatened taxa. This led some to argue that because conservation is a science of urgency, demographic and habitat changes are of more immediate importance than population genetics (Lande 1988; Caro and Laurenson 1994). In the last 45 years, we have seen a shift in the quantity and quality of genomic data, as well as necessary analytical methods, that are directly applicable to conservation (Kardos et al. 2021; Clancey et al. 2024). Genomics now informs many critical questions in conservation, ranging from evaluating adaptive potential to making decisions on translocations and genetic rescue (Shaffer et al. 2015). Although the debate over how to integrate genomics into on‐the‐ground conservation is still ongoing (Shafer et al. 2015; Hoban et al. 2023; Hogg 2024; Bertola et al. 2024), genomic data are now routinely used in decision‐making (Whiteley et al. 2015; Forester, Murphy, et al. 2022; Meek et al. 2023; Funk et al. 2012) and increasingly incorporated into conservation policy (Kershaw et al. 2022; Funk et al. 2019). The future of conservation will likely include ever more sophisticated applications of genomic resources as we manage biodiversity in our rapidly changing world (Allendorf et al. 2022).

Given the last 40 years of advances in integrating genomics into conservation, the challenge that remains is to focus our efforts on genomic resource coldspots. Our results suggest that the strategic path forward is to generate more genomic resources in the Global South, where biodiversity is high, but resources are comparatively low. This dovetails with a critical need for this work to be done equitably. Sustainable advancement of conservation genomics globally requires not only international collaborations between well‐resourced and under‐resourced countries but also local capacity building. This includes expanding training in molecular methods and bioinformatics, and redistribution of resources and financial support so that genomic studies can be led by Global South researchers (Asase et al. 2022; Barber et al. 2014; Gonzalez et al. 2023). Fortunately, successful models exist, including the ConGen workshop, which invites international participants and combines conceptual lectures with hands‐on analysis practice led by conservation genomics experts (Stahlke et al. 2020), the Nigerian Bioinformatics and Genomics Network, which fosters training of Nigerian researchers and facilitates international collaborations (Fatumo et al. 2020), and the Amphibian Genomics Consortium, an international effort to increase access to genomics knowledge and research collaborations (Kosch et al. 2024). An important role for Global North researchers is supporting these and other efforts, especially those led by Global South researchers. Finally, established Global North researchers should ensure their mentees are trained in developing equitable international collaborations, which will promote equity in the future and have cascading positive impacts on our research community (Haelewaters et al. 2021).

The field of conservation genomics has grown substantially, as has recognition that wildlife management is more effective with the integration of genomic data. The rise of new technologies, approaches, and bioinformatics pipelines has certainly contributed to this growth and promises to keep the pace of genomic studies high. Our review suggests that strategic development of genomic resources in the world's biodiversity hotspots should go hand‐in‐hand with provisioning of resources, funding, and capacity building for local scientists in these regions. This will maximise our success in this grand challenge for Earth's biodiversity.

## Author Contributions

We followed Civic Laboratory for Environmental Action Research (CLEAR)'s equity in author order guidelines to assign author order for this paper (Liboiron et al. 2017). C.M.C., A.S.‐E., A.E.B., T.X., R.L.W.A., C.M.A.‐L., M.P., J.J.J., H.C.T., E.W.B., K.R.Z., A.M.B.: designed and implemented the study. C.M.C., A.S.‐E., A.E.B., G.A.‐F., T.X., R.L.W.A., C.M.A.‐L., M.P., E.W.B., K.R.Z., A.M.B.: contributed to paper reviews and data extraction. A.S.‐E., A.E.B., G.A.‐F., T.X., J.J.J., H.C.T., A.M.B.: contributed to the design and implementation of figures and statistical analyses. C.M.C., A.S.‐E., A.E.B., G.A.‐F., T.X., R.L.W.A., C.M.A.‐L., J.J.J., H.C.T., K.R.Z., A.M.B.: contributed to writing for the first version of the manuscript. All authors read, edited, and approved the final version of the manuscript.

## Disclosure

Positionality Statement: We are a group of collaborators with national origins and recent ancestry spanning the Global North and the Global South. As a group, we have experience in international collaborations involving fieldwork in biodiversity conservation and research across North America, Central America, South America, Europe, the Caribbean, and Africa. We also represent multiple career stages and research expertise relevant to amphibian and reptile conservation and genomics. It is through our varied lenses of personal and professional identities that we present our perspectives and calls for advancement in global genomic resource sharing and capacity building.

## Conflicts of Interest

The authors declare no conflicts of interest.

## Benefit‐Sharing Statement

Benefits from this research accrue from the sharing of our data and results on public databases as described in Data Accessibility Statement.

## Supporting information


Appendix S1.

Appendix S2.

Appendix S3.

## Data Availability

All data included in this review can be found in Appendix S1, which is an Excel file containing all studies that were included in the Data Extraction phase, and all columns of data derived from those studies in Table 1; study counts by country in Table 2; relative amphibian richness (number of studies vs. per‐country richness) in Table 3; and relative reptile richness in Table 4. Supporting Information, systematic search parameters, and associated tables and figures are found in Appendix S2. Additional Supporting Information, tables and graphs are found in Appendix S3. Data and R scripts are also available at: https://github.com/gabferreira/Coldspots_in_genomic_resources [data to be uploaded upon acceptance].
