# Supplementary material for: Toward a Global Science of Conservation Genomics: Coldspots in Genomic Resources Highlight a Need for Equitable Collaborations and Capacity Building
Source: Mol Ecol. 2025 Mar 17;34(23):e17729. doi: 10.1111/mec.17729 (PMC12684328; doi:10.1111/mec.17729)
Supplement: Supplementary file 2 — Appendix S2. [file MEC-34-e17729-s002.pdf]

Supplemental File 2
Supplemental Methods; Tables S1-S3; Figures S1-S2

Supplemental Methods

Literature search and data filtering

We performed a comprehensive literature search in January 2024 using three databases: Web of Science (Core Collection), Agricola, and SciELO. For this search, we compiled a keyword list of general genetic and genomic terms, amphibian and reptile terms, adaptation, evolution or variation terms, and climate and environment terms (Table S1). The search was restricted to articles published in English as it is currently recognized as the international language of scientific publications, and therefore produces an efficient, albeit biased estimation of current relevant literature (Navarro et al. 2022; Suzina 2021; Tardy 2004). Keywords were matched based on article titles and abstracts for papers published in 2005-2024. We included “genetic” and “genomic” keywords in our search terms as well as names of specific genomic methodologies. The final list of amphibian and reptile terms consisted of common names and clades constructed by cross-referencing AmphibiaWeb, Amphibians of the World, The Reptile Database, and iNaturalist (AmphibiaWeb 2024; iNaturalist 2024; Frost 2024; Uetz et al. 2023). Papers located in our search were checked against 16 papers independently identified as relevant to ensure these were being captured with our search terms.

Data Extraction

After deduplicating papers, we imported them into Rayyan (Ouzzani et al. 2016) for two rounds of filtering by coauthors (Fig. S1). In the first round, titles and abstracts were independently reviewed by two randomly assigned coauthors to assess whether they met two inclusion criteria: (1) the study species were amphibians and/or reptiles and (2) the study generated genomic level data (Table S2). We defined genomic data as any genome-scale assessment of variation and excluded papers that generated exclusively mitochondrial or microsatellite data. Papers were excluded if they did not meet both criteria. If this information was unobtainable from the title and abstract, we included the paper and tagged it as “maybe genomic” (Fig. S2). Papers selected for inclusion by both reviewers underwent a second round of filtering and data extraction. Papers included by one coauthor but excluded by a second coauthor were checked by a third randomly assigned reviewer to determine if the study met eligibility criteria.

Genomic data type was categorized as “RNAseq”, “GBS/RAD/target capture”, or “whole genome.” Level 1 (general genomic resource) studies included a reference genome or transcriptome without any spatial or functional analysis, or studies of the genomic architecture of particular traits (e.g., genetics of sex determination or functional genomics of venom glands). Level 2 (spatial genomic variation) studies characterized genomic variation among populations within species, often including some demographic history and/or phylogeographic analyses. Level 3 (functional variation - global change) studies reported on the genomic underpinnings of organismal responses to stressors related to global change, but not specifically to climate change. These studies often identified loci under selection or differential gene expression in response to stressors such as disease, UV exposure, or spread of introduced species. Level 4

(functional variation - climate change) studies also reported on the genomic underpinnings of organismal responses, but specifically focused on factors relevant to climate change, including temperature, precipitation/drought, or seasonality. Finally, Level 5 (adaptive potential) studies explicitly used genomics to examine organismal responses and vulnerability to future climate change using experimental approaches such as common garden experiments or forecasting models based on genomic data combined with climate change projections. We included studies of both wild animals and captive animals (i.e., animals from zoos, laboratory colonies, or farms) in our dataset, but for captive animals we extracted data only on species identity, as they were not assignable to a native geographic location. For studies of wild animals, we extracted data on species identity, sample geographic locality data, and author information (Table S3).

References

AmphibiaWeb. (2024). <https://amphibiaweb.org> University of California, Berkeley, CA, USA. Accessed 29 November 2023.

Frost, D. R. (2024). Amphibian Species of the World: an online reference. Version 6.2. <http://research.amnh.org/vz/herpetology/amphibia/> Accessed 29 November 2023.

iNaturalist. (2024). <https://www.inaturalist.org> Accessed 29 November 2023.

Navarro, F., Lillis, T., Donahue, T., Curry, M. J., Reyes, N. Á., Gustafsson, M., Zavala, V., Lauría, D., Lukin, A., McKinney, C., Feng, H., & Motta-Roth, D. (2022). Rethinking English as a lingua franca in scientific-academic contexts. Journal of English for Research Publication Purposes, 3(1), 143–153.

Ouzzani, M., Hammady, H., Fedorowicz, Z., & Elmagarmid, A. (2016). Rayyan—a web and mobile app for systematic reviews. Systematic Reviews, 5(1), 210.

Suzina, A. C. (2021). English as lingua franca. Or the sterilisation of scientific work. Media Culture & Society, 43(1), 171–179.

Tardy, C. (2004). The role of English in scientific communication: lingua franca or Tyrannosaurus rex? Journal of English for Academic Purposes, 3(3), 247–269.

Uetz, P., Freed, P, Aguilar, R., Reyes, F., Kudera, J. & Hošek, J. (eds.). (2023). The Reptile Database. <http://www.reptile-database.org> Accessed 12 July 2024.

Table S1. Complete list of search terms, including overall results and database-specific search formats. Final search was completed on January 16, 2024.

Genomic	gene OR genes OR genetic* OR genom*
Study Focus	adapt* OR differen* OR compari* OR evolution* OR varia*
Species	Adder OR Agama OR Alligator OR Amphibia* OR Amphisbaenian OR Amphiuma OR Anole OR Anura OR Apoda OR Archaebatrachia OR Asp OR Axolotl OR Baskilisk OR Blindsnake OR Boa OR Caecilian OR Caiman OR Caudata OR Chameleon OR Chelonia* OR Chuckwalla OR Coachwhip OR Cobra OR Cooter OR Crocodile OR Crocodilia* OR Cryptodira OR Dragon OR Ensatina OR Frog OR Galliwasps OR Gecko OR Gharial OR “Gila monster” OR Goanna OR Gymophiona OR Hellbender OR Herpet* OR Krait OR Lizard OR Mamba OR Mamushi OR Matamata OR Mesobatrachia OR Mudpuppy OR Neobatrachia OR Newt OR Olm OR Pleurodira OR Pobblebonk OR Python OR Racer OR Racerunner OR Reptilia* OR Rhino OR Salamander OR Salientia OR Sauria OR Serpent OR Serpentes OR Siren OR Skink OR Slider OR Snake OR Spadefoot OR squamata OR squeeker OR Tegu OR Terrapin OR Testudines OR Toad OR Tortoise OR Treefrog OR Tuatara OR Turtle OR Urodela OR Viper OR “Water dog” OR Whiptail
Context	Altitud* OR “Climate change” OR Elevation* OR Environment* OR Geograph* OR Population* OR Thermal*

Results for each database queried with these search terms:

Web of Science: Core Collection: 8,915
Agricola: 3,490
SciELO: 145

Searches conducted within each database broken down on the following pages with exact formatting provided for each search string within all three databases.

Web of Science: Core Collection; Date searched: January 16, 2024; Exact Search: ON

Search line number	Search string
7	#5 AND #6 and Article or Early Access or Data Paper (Document Types)
6	DOP=(2005/2024)
5	#1 AND #2 AND #3 AND #4
4	TS=(Altitud* OR “Climate change” OR Elevation* OR Environment* OR Geograph* OR Population* OR Thermal*)
3	TS=(Adder OR Agama OR Alligator OR Amphibia* OR Amphisbaenian OR Amphiuma OR Anole OR Anura OR Apoda OR Archaebatrachia OR Asp OR Axolotl OR Baskilisk OR Blindsnake OR Boa OR Caecilian OR Caiman OR Caudata OR Chameleon OR Chelonia* OR Chuckwalla OR Coachwhip OR Cobra OR Cooter OR Crocodile OR Crocodilia* OR Cryptodira OR Dragon OR Ensatina OR Frog OR Galliwasps OR Gecko OR Gharial OR “Gila monster” OR Goanna OR Gymophiona OR Hellbender OR Herpet* OR Krait OR Lizard OR Mamba OR Mamushi OR Matamata OR Mesobatrachia OR Mudpuppy OR Neobatrachia OR Newt OR Olm OR Pleurodira OR Pobblebonk OR Python OR Racer OR Racerunner OR Reptilia* OR Rhino OR Salamander OR Salientia OR Sauria OR Serpent OR Serpentes OR Siren OR Skink OR Slider OR Snake OR Spadefoot OR squamata OR squeeker OR Tegu OR Terrapin OR Testudines OR Toad OR Tortoise OR Treefrog OR Tuatara OR Turtle OR Urodela OR Viper OR “Water dog” OR Whiptail)
2	TS=(adapt* OR differen* OR compari* OR evolution* OR varia*)
1	TS=(Gene OR genes OR genetic* OR Genom*)

Agricola (EBSCO interface)
Date searched: January 16, 2024
Search modes - Boolean/Phrase, Apply Equivalent Subjects: OFF, Apply Related Words: OFF

Search line number	Search string
S6	(S1 AND S2 AND S3 AND S4) ADD Limiters: 20050101-20231231
S5	(S1 AND S2 AND S3 AND S4)
S4	Altitud* OR “Climate change” OR Elevation* OR Environment* OR Geograph* OR Population* OR Thermal*
S3	Adder OR Agama OR Alligator OR Amphibia* OR Amphisbaenian OR Amphiuma OR Anole OR Anura OR Apoda OR Archaebatrachia OR Asp OR Axolotl OR Baskilisk OR Blindsnake OR Boa OR Caecilian OR Caiman OR Caudata OR Chameleon OR Chelonia* OR Chuckwalla OR Coachwhip OR Cobra OR Cooter OR Crocodile OR Crocodilia* OR Cryptodira OR Dragon OR Ensatina OR Frog OR Galliwasps OR Gecko OR Gharial OR “Gila monster” OR Goanna OR Gymophiona OR Hellbender OR Herpet* OR Krait OR Lizard OR Mamba OR Mamushi OR Matamata OR Mesobatrachia OR Mudpuppy OR Neobatrachia OR Newt OR Olm OR Pleurodira OR Pobblebonk OR Python OR Racer OR Racerunner OR Reptilia* OR Rhino OR Salamander OR Salientia OR Sauria OR Serpent OR Serpentes OR Siren OR Skink OR Slider OR Snake OR Spadefoot OR squamata OR squeeker OR Tegu OR Terrapin OR Testudines OR Toad OR Tortoise OR Treefrog OR Tuatara OR Turtle OR Urodela OR Viper OR “Water dog” OR Whiptail
S2	adapt* OR differen* OR compari* OR evolution* OR varia*
S1	Gene OR genes OR genetic* OR Genom*

SciELO (Web of Science Interface); Date searched: January 16, 2024; Exact Search: ON

Search line number	Search string
7	#5 AND #6 and Editorial or Review Article (Exclude – Document Types)
6	PY=(2005/2024)
5	#1 AND #2 AND #3 AND #4
4	TS=(Altitud* OR “Climate change” OR Elevation* OR Environment* OR Geograph* OR Population* OR Thermal*)
3	TS=(Adder OR Agama OR Alligator OR Amphibia* OR Amphisbaenian OR Amphiuma OR Anole OR Anura OR Apoda OR Archaebatrachia OR Asp OR Axolotl OR Baskilisk OR Blindsnake OR Boa OR Caecilian OR Caiman OR Caudata OR Chameleon OR Chelonia* OR Chuckwalla OR Coachwhip OR Cobra OR Cooter OR Crocodile OR Crocodilia* OR Cryptodira OR Dragon OR Ensatina OR Frog OR Galliwasps OR Gecko OR Gharial OR “Gila monster” OR Goanna OR Gymophiona OR Hellbender OR Herpet* OR Krait OR Lizard OR Mamba OR Mamushi OR Matamata OR Mesobatrachia OR Mudpuppy OR Neobatrachia OR Newt OR Olm OR Pleurodira OR Pobblebonk OR Python OR Racer OR Racerunner OR Reptilia* OR Rhino OR Salamander OR Salientia OR Sauria OR Serpent OR Serpentes OR Siren OR Skink OR Slider OR Snake OR Spadefoot OR squamata OR squeeker OR Tegu OR Terrapin OR Testudines OR Toad OR Tortoise OR Treefrog OR Tuatara OR Turtle OR Urodela OR Viper OR “Water dog” OR Whiptail)
2	TS=(adapt* OR differen* OR compari* OR evolution* OR varia*)
1	TS=(Gene OR genes OR genetic* OR Genom*)

Table S2. PECO diagram. Framework used to identify questions for this study and inclusion/exclusion criteria (Foo et al. 2021; Morgan et al. 2018).

**Project Component**	**Inclusion**	**Exclusion**
**Population:** Herpetofauna (reptiles and amphibians)	Articles on wild and captive individuals and/or populations of herpetofauna	Articles that do not include herpetofauna
**Exposure:**<br>1) Lack of genomic resources for herpetofauna relative to species richness (cold spot 1).<br><br>2) Lack of genomic research on adaptive potential of herps relative to predicted environmental change (cold spot 2).	Articles that generate genomic data	Articles that generate mitochondrial or microsatellite data
**Comparator:** Geographic region of study (across space)	Articles from any geographic location	
**Outcomes:** Identify the two types of “cold spots”		

Table S3: All data extraction fields with criteria adopted for each variable.

Column Header	Description
Study ID	Unique identifying number assigned to each study.
Author(s)	List of all authors of study (Last Name, First Name, Middle Initial).
Year	Year of Publication.
Title	Publication Title.
Journal	Publication Journal (as exported from Rayyan).
Scope	Selection Options: Level 1 - general genomic resource, Level 2 - spatial variation, Level 3 - Functional genomic variation: global change, Level 4 - Functional genomic variation: climate change, Level 5 - adaptive potential - climate change. Scope of the study based on the relevance of genomic data and analyses to global change and/or climate change.
Genomic	Selection Options: RNASeq (including traditional RNASeq, RNA microarrays, and transcriptomics), GBS/RAD/target capture (any reduced representation approach), whole genome (including low coverage and genome resequencing). Type of genomic data is collected as part of this study. If multiple data types are collected an additional row was added to the study.
Wild or Captive	Selection Options: Wild or Captive. Identifies if individuals/populations studied are wild-caught or captive. If both are present, an additional row was added to the study. If captive is selected, only latin binomial, class, IUCN latin binomial and IUCN status (IUCN 2024) were filled out and all other remaining columns were left blank.
Continent	Selection Options: North America, South America, Oceania, Europe, Asia, Africa. Continent where the genomic work took place. Continent assignment is based on the tectonic plate where sampling occurred. Continent assignment for countries that span multiple continents was based on where genomic work took place.
Country	Country where samples were collected. If multiple countries were sampled in the same study, an additional row was added to the same study.

Column Header	Description
Latitude	Latitude for study sites. If one latitude coordinate was provided it was directly copied from the study. If multiple were provided they were averaged for a single point per country. For studies with multiple species or countries additional rows were added for the same study to reflect these additional localities. If specific coordinates were not provided but there were country or specific location details noted either in text or in figures, coordinates were identified from a central point on Google Maps. If no locality information was provided, coordinates were determined from averaging a location across the species range (IUCN 2024). All coordinates were converted to decimal degrees if not originally provided in that format.
Longitude	Longitude for study sites. If one longitude coordinate was provided it was directly copied from the study. If multiple were provided they were averaged for a single point per country. For studies with multiple species or countries additional rows were added for the same study to reflect these additional localities. If specific coordinates were not provided but there were country or specific location details noted either in text or in figures, coordinates were identified from a central point on Google Maps. If no locality information was provided, coordinates were determined from averaging a location across the species range (IUCN 2024). All coordinates were converted to decimal degrees if not originally provided in that format.
Avg or Est Points	Selection Options: Yes or No. Yes indicates that the lat/long coordinates provided are an average or estimation of the location of samples in the study.
Coordinate Notes	Any additional information about how coordinates were located, averaged or estimated.
Coordinate System	Selection Options: DD, UTM, DMS, DDM, NA. Corresponds to the coordinate format noted in the lat/long columns. (DD = Decimal Degrees, UTM = Universal Transverse Mercator, DMS = Degrees Minutes Seconds, DDM = Degrees Decimal Minutes).
Class	Selection Options: Amphibia or Reptilia. Class for the species reported in the study.

Column Header	Description
Latin Binomial	Latin binomial for the species present in the study. If there were multiple species in the study, additional rows were added to the same study. Subspecies were not considered as separate species, and any species that were not identified or not yet described (those with "sp." instead of a species epithet) or noted as a taxonomic uncertainty (cf. listed between the genus and species) were excluded.
IUCN Latin Binomial	Latin binomial for the species as identified in IUCN database (IUCN 2024).
IUCN Status	IUCN Status (LC = Least Concern, VU = Vulnerable, NT = Near Threatened, EN = Endangered, CR = Critically endangered, EX = Extinct, EW = Extinct in Wild, DD = Data Deficient, NA = not in IUCN database (IUCN 2024).
Species Global North or Global South	Country where this species is located (from the study) is in the Global North or Global South. Countries were used to determine whether sampling occurred in the Global North or Global South; these regions were defined based on the United Nations Conference on Trade and Development (UNCTAD 2023).
Total Number of Authors	Total number of authors for the publication.
1st Author Origin Country	Origin country for the first author based on the first affiliated institution.
1st Author Global Divide	Country of primary institution affiliation for the 1st author is in the Global South or Global North the United Nations Conference on Trade and Development (UNCTAD 2023).
Last Author Global Divide	Country of primary institution affiliation for the last author is in the Global South or Global North the United Nations Conference on Trade and Development (UNCTAD 2023).
Last Author Origin Country	Origin country for the senior author based on the first affiliated institution.
Collaboration	Selection Options: Single Author, Domestic or International. Single Author if there is only one author for the publication; Domestic if all the authors' primary institution affiliation are in the same country; International if the authors' primary institution affiliation are in more than one country.
Number of Local Authors	Number of Local Authors. An author is considered local if their primary institution affiliation is in a country where at least one of the species in the study is sampled from.

Column Header	Description
Number of Authors from Global North	Number of Authors whose primary institution affiliation is a Global North country the United Nations Conference on Trade and Development (UNCTAD 2023).
Number of Authors from Global South	Number of Authors whose primary institution affiliation is a Global South country the United Nations Conference on Trade and Development (UNCTAD 2023).
delta_maximum_temperature_1960_2050	Maximum expected change in temperature (°C) predicted to occur from 1960-2050 at the coordinates inferred for the study.
Paper Notes	Any notes about genomic data, territories of countries, scope or authors for future reference.

Figure S1. PRISMA diagram (Page et al. 2021) showing the number of records identified from the database, records screened, records excluded during first and second screening, and the total number included in the study.

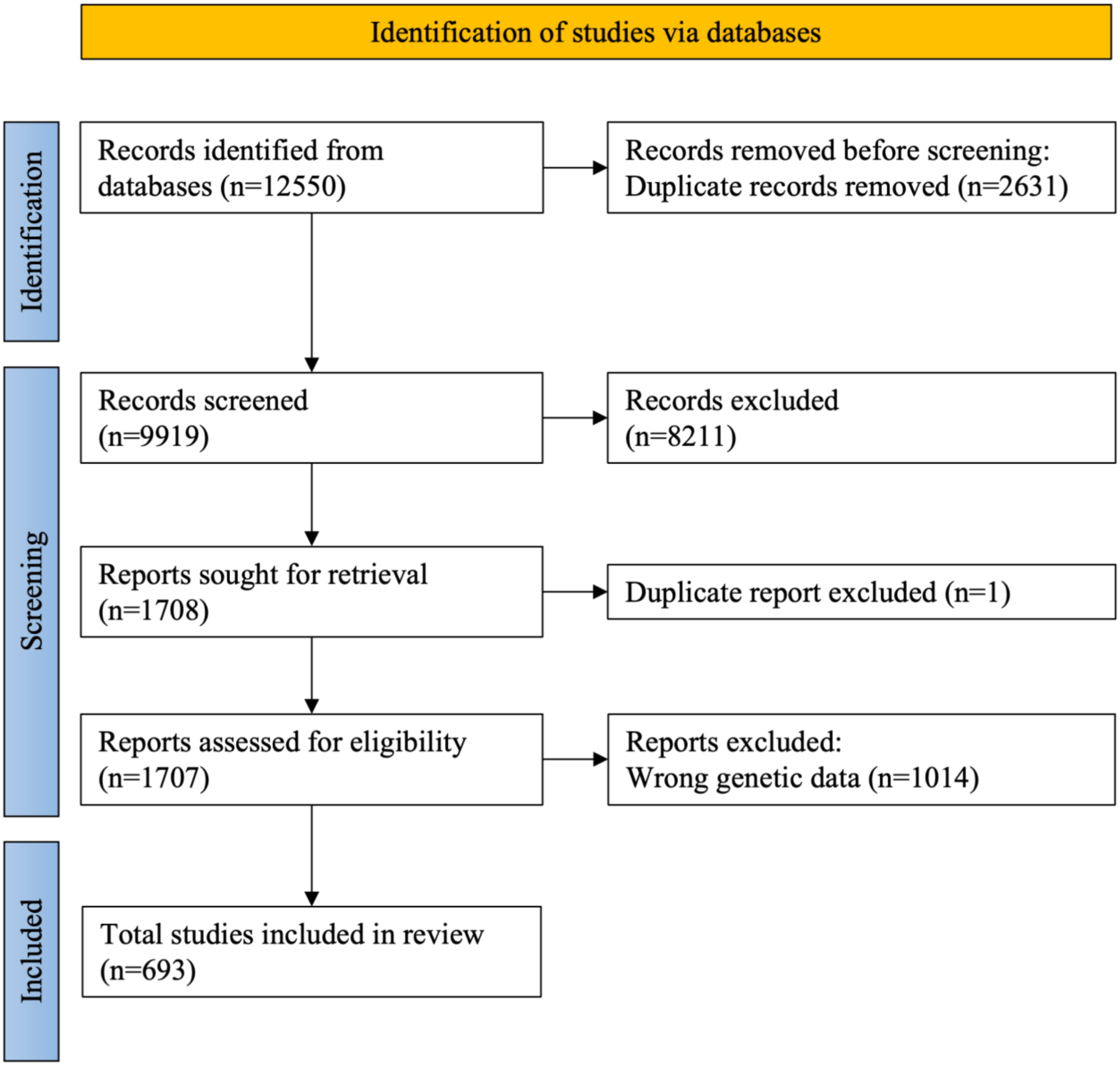

Figure S2. Genomic decision tree and genomic terms used in Round 1 of filtering. We used this decision tree, and a list of common genomic terms, as a guideline for inclusion or exclusion of papers.

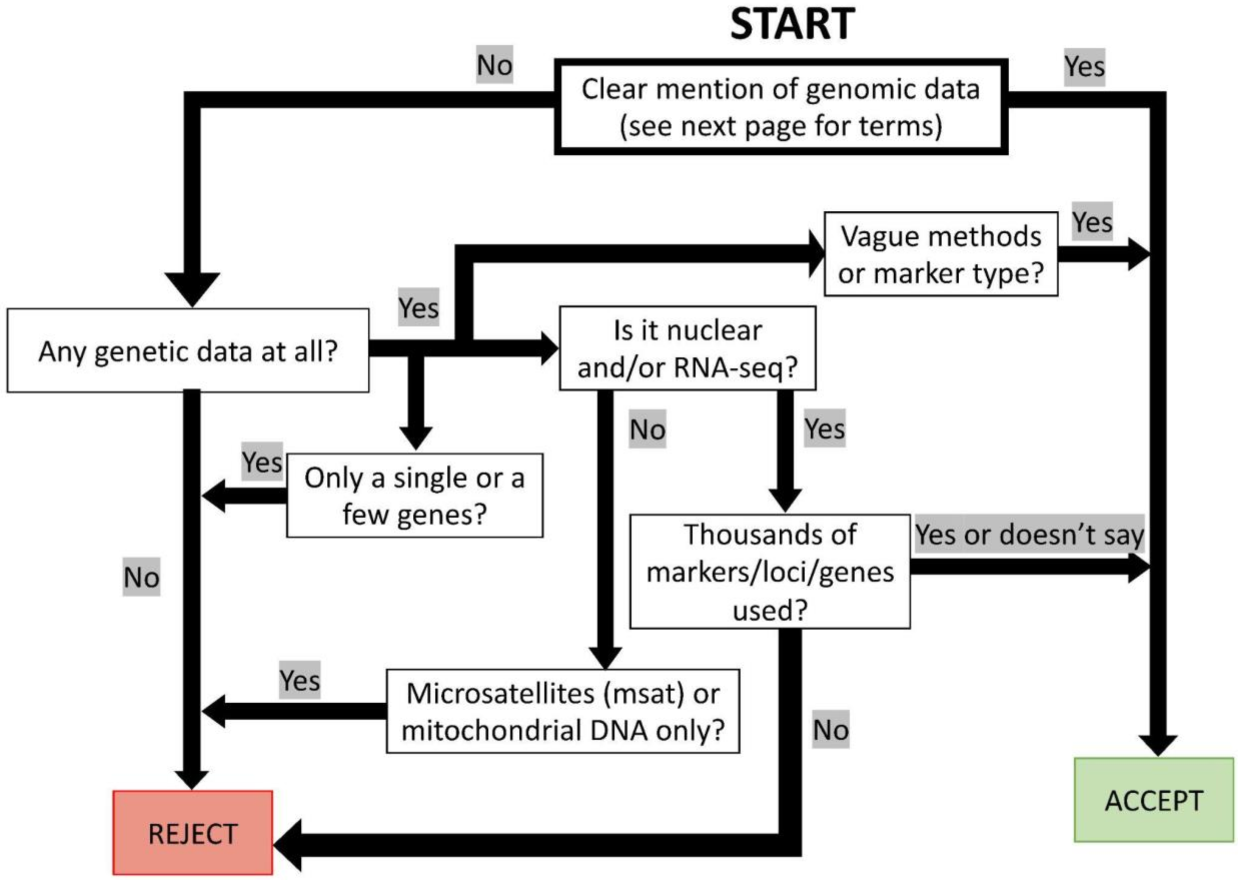

Common Genomic Terms: SNP, SNP array, SNP chip, Target capture, RNA sequence, RNA sequencing, RNA-seq/RNAseq, Transcriptomics, Transcriptome, Genome sequence, Differential gene expression, Gene expression, reduced representation sequencing, RAD-seq/RADseq, RADsequence, Reference genome, Ultraconserved elements, Anchored hybrid enrichment, 2bRAD, GBS, ddRAD, genotype by sequencing, whole genome, whole genome sequencing, genome resequencing, sequence capture, exome capture, exon capture, whole exome sequencing, exome sequencing, exon sequencing, 3RAD, Restriction site associated DNA sequencing, Hybridization capture, Next generation sequencing, NGS, Long read sequencing, third generation sequencing, shotgun sequencing

References

Foo, Y. Z., O'Dea, R. E., Koricheva, J., Nakagawa, S., & Lagisz, M. (2021). A practical guide to question formation, systematic searching and study screening for literature reviews in ecology and evolution. *Methods in Ecology and Evolution / British Ecological Society*, *12*(9), 1705–1720.

IUCN. (2024). The IUCN Red List of Threatend Species. <https://www.iucnredlist.org> Accessed 27 June 2024.

Morgan, R. L., Whaley, P., Thayer, K. A., & Schünemann, H. J. (2018). Identifying the PECO: A framework for formulating good questions to explore the association of environmental and other exposures with health outcomes. *Environment International*, *121*(Pt 1), 1027–1031.

Page, M. J., McKenzie, J. E., Bossuyt, P. M., Boutron, I., Hoffmann, T. C., Mulrow, C. D., Shamseer, L., Tetzlaff, J. M., Akl, E. A., Brennan, S. E., Chou, R., Glanville, J., Grimshaw, J. M., Hróbjartsson, A., Lalu, M. M., Li, T., Loder, E. W., Mayo-Wilson, E., McDonald, S., … Moher, D. (2021). The PRISMA 2020 statement: an updated guideline for reporting systematic reviews. *BMJ* , *372*, n71.

UNCTAD. (2023). *Handbook of Statistics 2023 - Classification of Global Economies*. United Nations Conference on Trade and Development. https://hbs.unctad.org/classifications/
