# Supplementary material for: Toward a Global Science of Conservation Genomics: Coldspots in Genomic Resources Highlight a Need for Equitable Collaborations and Capacity Building
Source: Mol Ecol. 2025 Mar 17;34(23):e17729. doi: 10.1111/mec.17729 (PMC12684328; doi:10.1111/mec.17729)
Supplement: Supplementary file 3 — Appendix S3. [file MEC-34-e17729-s003.pdf]

Supplemental File 3

Tables S4-S11; Figures S3-S10

Table S4. Genomic resources in five scope categories (ranked according to their applicability to conservation under global change) and their distribution across studies of captive and wild amphibians and reptiles.

Scope	Wild	Captive	Grand Total
Level 1: General Genomic Resource	194	66	252
Level 2: Spatial Genomic Variation	318	4	320
Level 3: Functional Variation - Global Change	48	18	64
Level 4: Functional Variation - Climate Change	46	6	51
Level 5: Adaptive Potential	14	4	16
**Grand Total**	**610**	**98**	**693**

Table S5. Number of studies reporting different kinds of genomic data (reduced representation data, functional genomics, or whole genomes) and their distribution across studies of wild amphibians and reptiles.

Genomic	Amphibia	Reptilia	Total
GBS/RAD/Target Capture	186	216	400
RNASeq	138	109	247
Whole Genome	16	60	76
**Grand Total**	**330**	**365**	**693**

Table S6. Number of species for which we identified genomic resources (reduced representation data, functional genomics, or whole genomes) and their distribution across studies of wild amphibians and reptiles.

Genomic	Amphibia	Reptilia	Grand Total
GBS/RAD/Target Capture	479	668	1147
RNASeq	119	134	253
Whole Genome	27	129	156
**Grand Total**	**557**	**825**	**1382**

Table S7. Number of studies and species (wild only) in our dataset by Global South and Global North.

Count (Wild Only)	Amphibia	Reptilia	Total
Global North Studies	184	207	390
Global South Studies	131	155	285
N/A	1	4	5
**All Studies (Wild Only)**	**291**	**321**	**610**
Global North Species	202	423	625
Global South Species	372	379	751
N/A	2	9	11
**All Species (Wild Only)**	**552**	**774**	**1326**

Table S8. Pooled reptile and amphibian genomic resources in five scope categories (ranked according to their applicability to conservation under global change) and the frequency of different kinds of data collected in those scope categories.

Scope	GBS/RAD/ Target Capture	RNASeq	Whole Genome	Grand Total
Level 1: General Genomic Resource	57	154	57	252
Level 2: Spatial Genomic Variation	299	18	11	320
Level 3: Functional Variation - Global Change	15	46	4	64
Level 4: Functional Variation - Climate Change	28	20	4	51
Level 5: Adaptive Potential	5	11	0	16
**Grand Total**	**400**	**247**	**76**	**693**

Table S9. Number of reptile and amphibian species, by IUCN threat categories, for which we recovered genomic resources in any scope category in our review. The total number of species of reptiles and amphibians combined exceeds the number of publications in our review because many publications included genomic resources for more than one species.

IUCN Status	Amphibia	Reptilia	Grand Total
LC	372	561	933
NT	26	32	58
VU	42	44	86
EN	43	37	80
CR	26	25	51
EW	1	2	3
EX	2	2	4
DD	10	23	33
NA	36	102	138
**Grand Total**	**557**	**825**	**1382**

Table S10. Attributes of genomics studies of wild amphibians and reptiles by continent of sampling. Studies with local authors had at least one author from the country of sampling; domestic collaborations included all local authors, while international collaborations included authors from multiple countries; first and last author global divide (Global North vs. Global South) was assessed based on authors' primary institutional affiliations listed on the publication; genomic resources are defined as unique combinations of study identity, species, scope, and country of sampling. Column sums exceed the total number of studies because some studies include sampling in multiple continents, and many studies include multiple genomic resources.

Continent	Total # studies	Local Authors	Domestic Collaboration	International Collaboration	First Author Global North	First Author Global South	Last Author Global North	Last Author Global South	Total # Genomic Resources
Africa	43	29	5	38	36	7	39	4	198
Asia	146	137	71	75	58	89	66	81	491
Europe	108	88	28	80	99	9	107	1	473
North America	242	234	159	83	225	19	228	16	693
Oceania	71	71	42	29	68	3	69	2	345
South America	69	57	15	54	44	24	55	16	288

Table S11. Number of studies for international and domestic collaboration, and whether the first author is from the Global North or Global South across studies of wild amphibians and reptiles. The total number of species of reptiles and amphibians combined exceeds the number of publications in our review because some publications included both reptiles and amphibians.

Count (Wild Only)	Amphibia	Reptilia	Grand Total
Domestic Collaboration Studies	159	179	338
International Collaboration Studies	149	166	315
Studies with First Author from the Global North	219	251	470
Studies with First Author from the Global South	72	70	142
Studies with Last Author from the Global North	234	263	497
Studies with Last Author from the Global South	57	58	115
**Grand Total**	**599**	**666**	**1265**

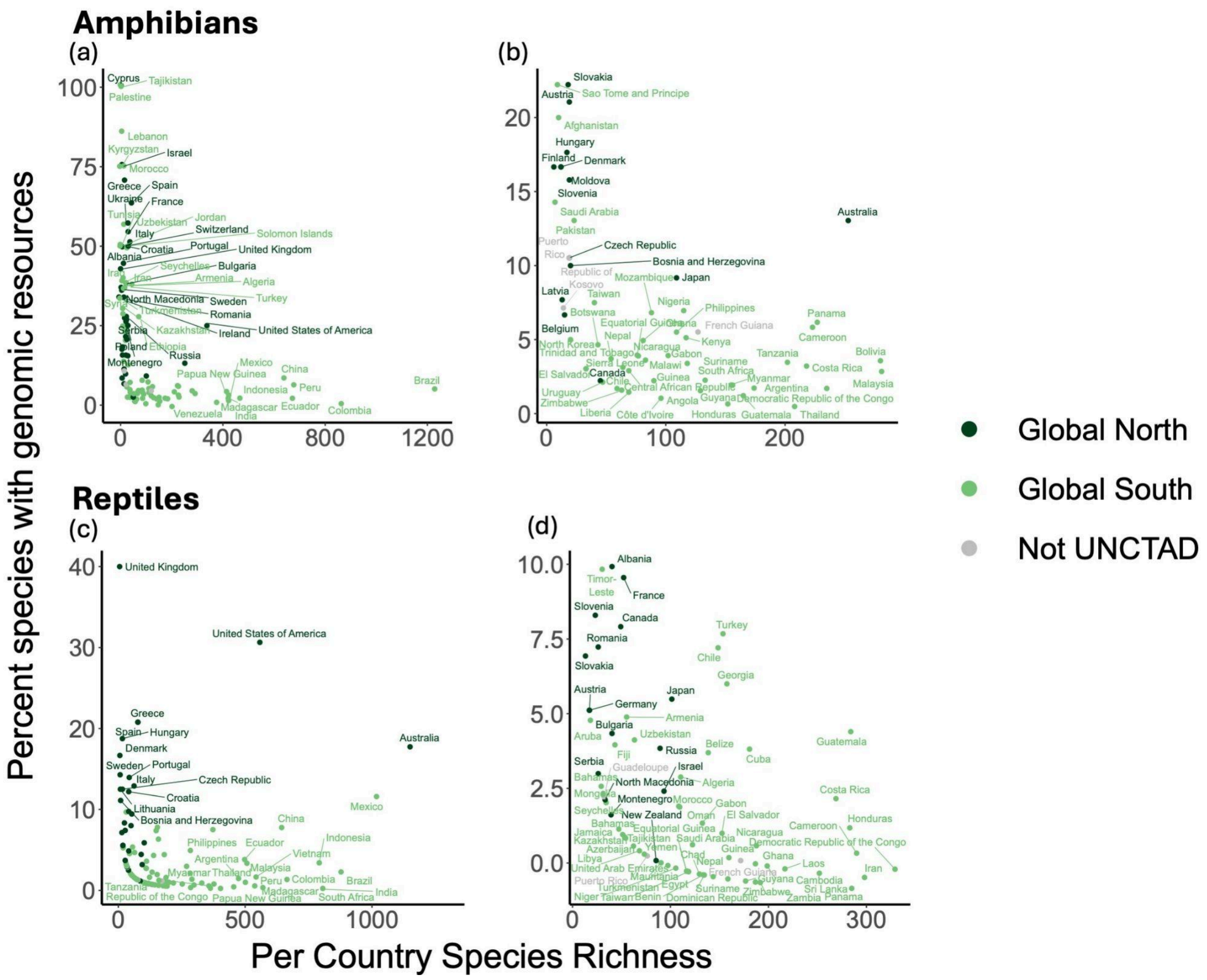

Figure S3. Percentage of species with published genomic resources over species richness per country for amphibians (a-b) and reptiles (c-d). Country points are labeled and colored according to the United Nations Conference on Trade and Development (UNCTAD) names and designations, with countries not appearing in the UNCTAD list coded as “Not UNCTAD” (gray).

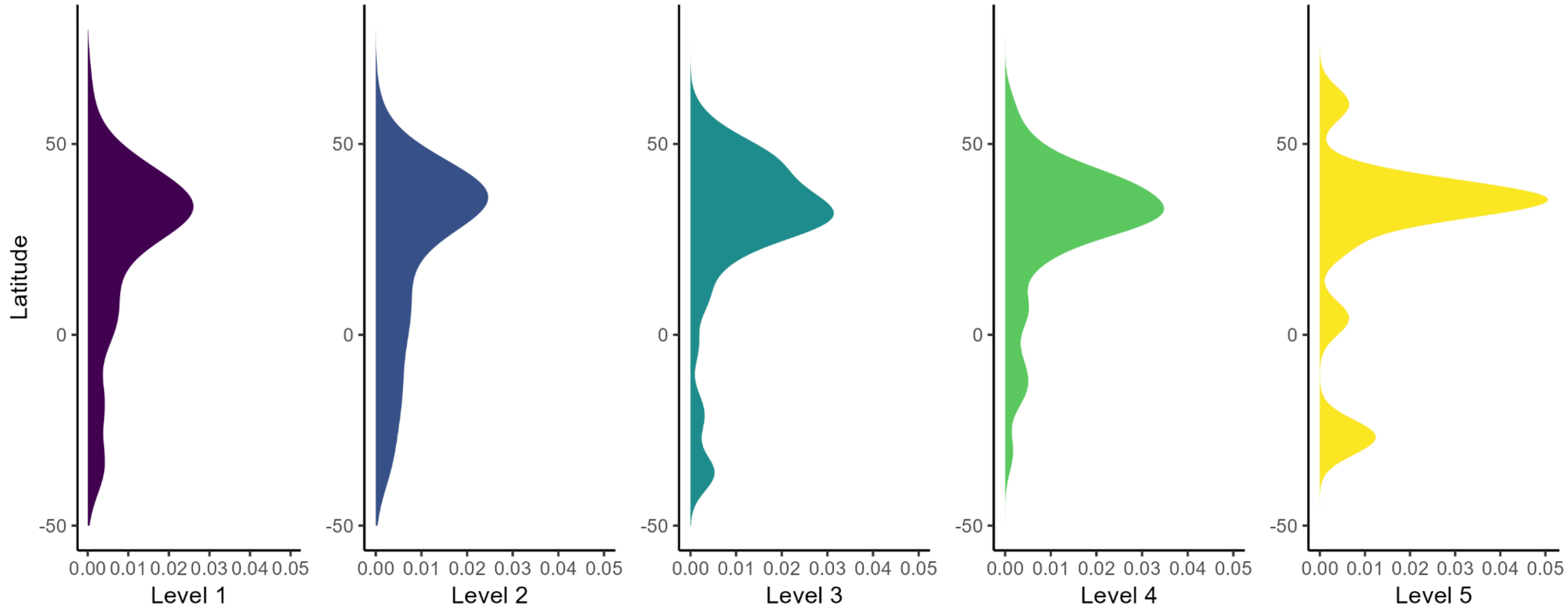

Figure S4. Density plots of published genomic resources by genomic scope level ordered by latitude (n study = 693; n genomic resources = 703). Increasing scope level indicates increasing applicability to conservation in the face of global change; see text for additional details. Genomic resources become more scarce and concentrated at higher latitudes as genomic scope increases.

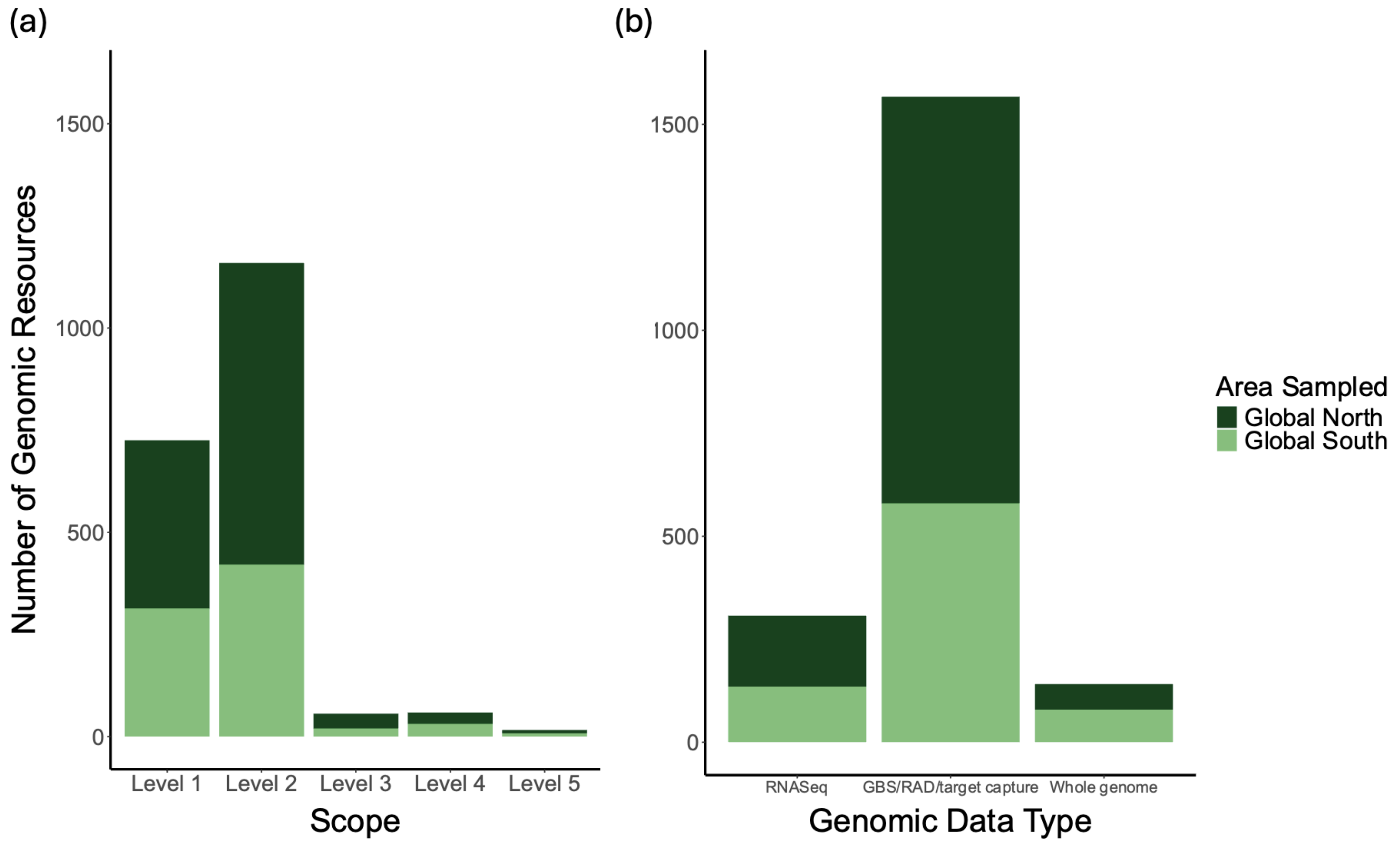

Figure S5. Number of genomic resources by the global region sampled (Global North or Global South) and (a) genomic scope level or (b) genomic data type (n studies = 609, n genomic resources = 2015). A genomic resource is defined as a unique combination of study identity, scope, genomic data, and global region combination for a particular species.

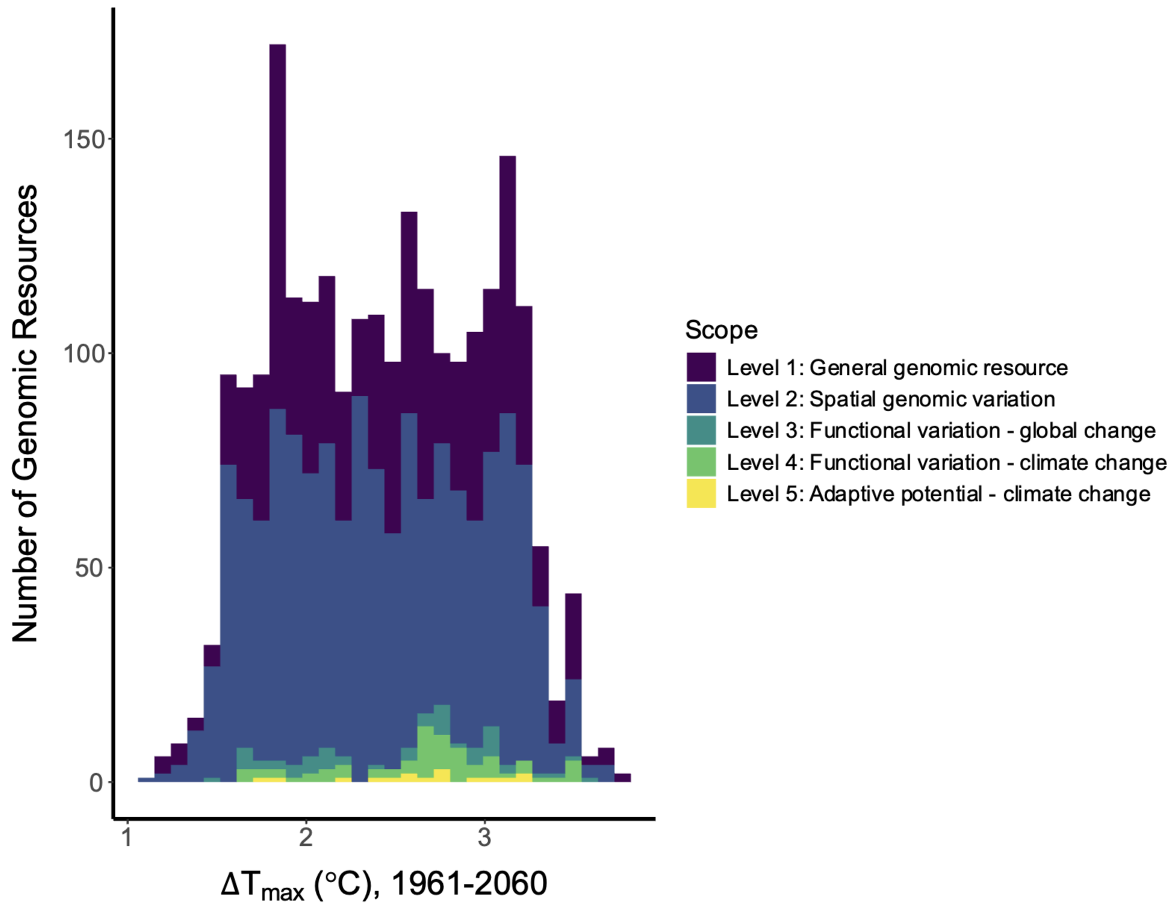

Figure S6. Histogram of the number of published genomic resources in the five genomic scope levels by predicted overall change in maximum temperature (ΔT_{max} [°C] 1961-2060) for each study's sampling location (n studies = 579; n genomic resources = 2323). Larger ΔT_{max} values represent regions expected to show the highest challenge to ectotherm persistence, and those areas in particular would benefit from additional genomic resources with higher applicability to predicting organismal responses (Levels 3-5). The majority of genomic resources spanning the range of ΔT_{max} values are Levels 1-2.

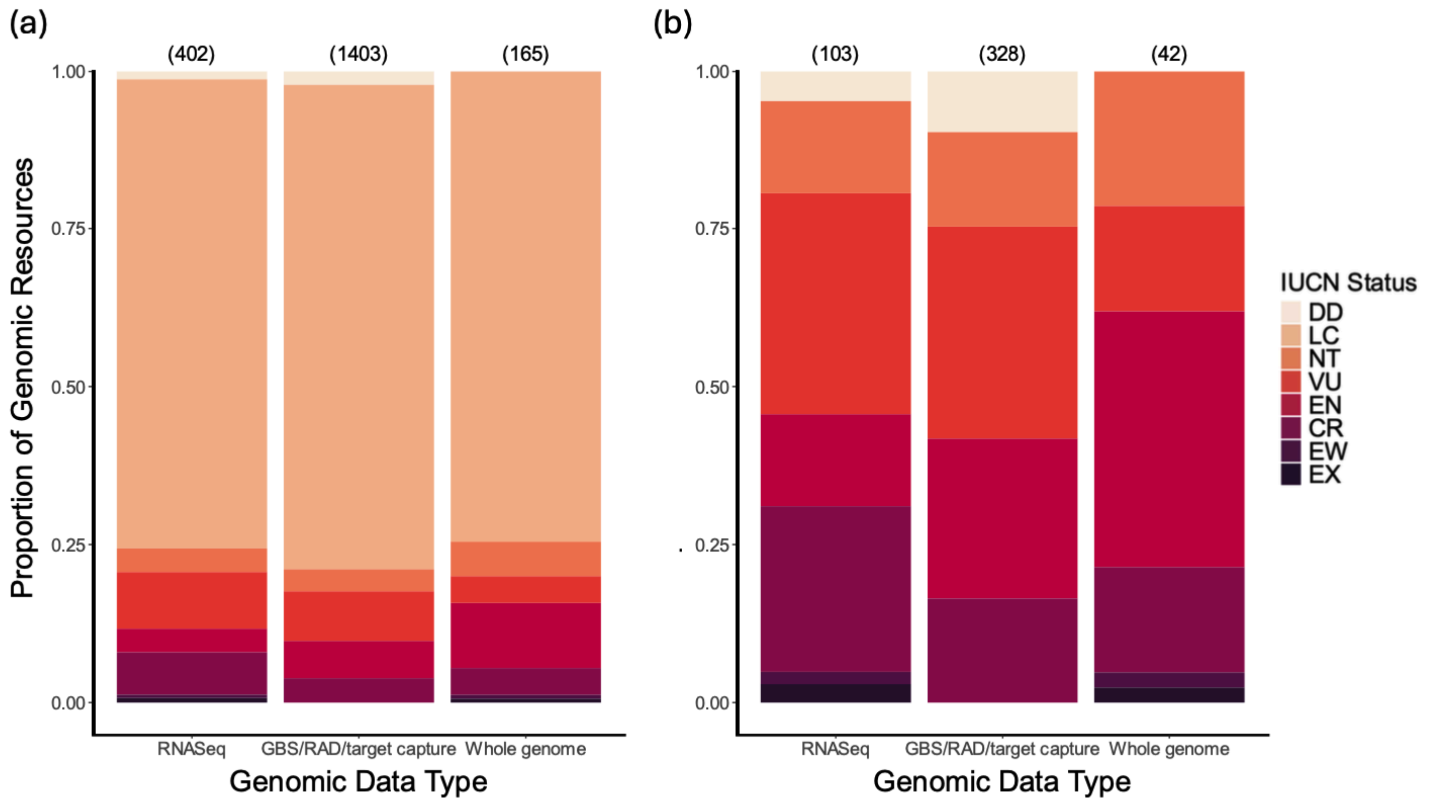

Figure S7. Proportion of genomic resources grouped by genomic data type and IUCN threat categories for species with IUCN data available, excluding data deficient (DD) species. Genomic resources here are defined as a unique combination of study identity, scope and genomic data type for a particular species. Data types include RNASeq (transcriptome, RNA microarrays, and traditional RNA sequencing), GBS/RAD/target capture (all reduced representation approaches using gDNA); and whole genome (including low coverage and genome resequencing). (a) Proportion of genomic resources of each data type for species of all threat categories (n studies = 672, n genomic resources = 1933). (b) Proportion of genomic resources of each data type for species of all threat categories except least concern (LC) (n studies = 235, n genomic resources = 436). Numbers above bars represent the total number of genomic resources in each genomic scope category. Wild and captive animals were included.

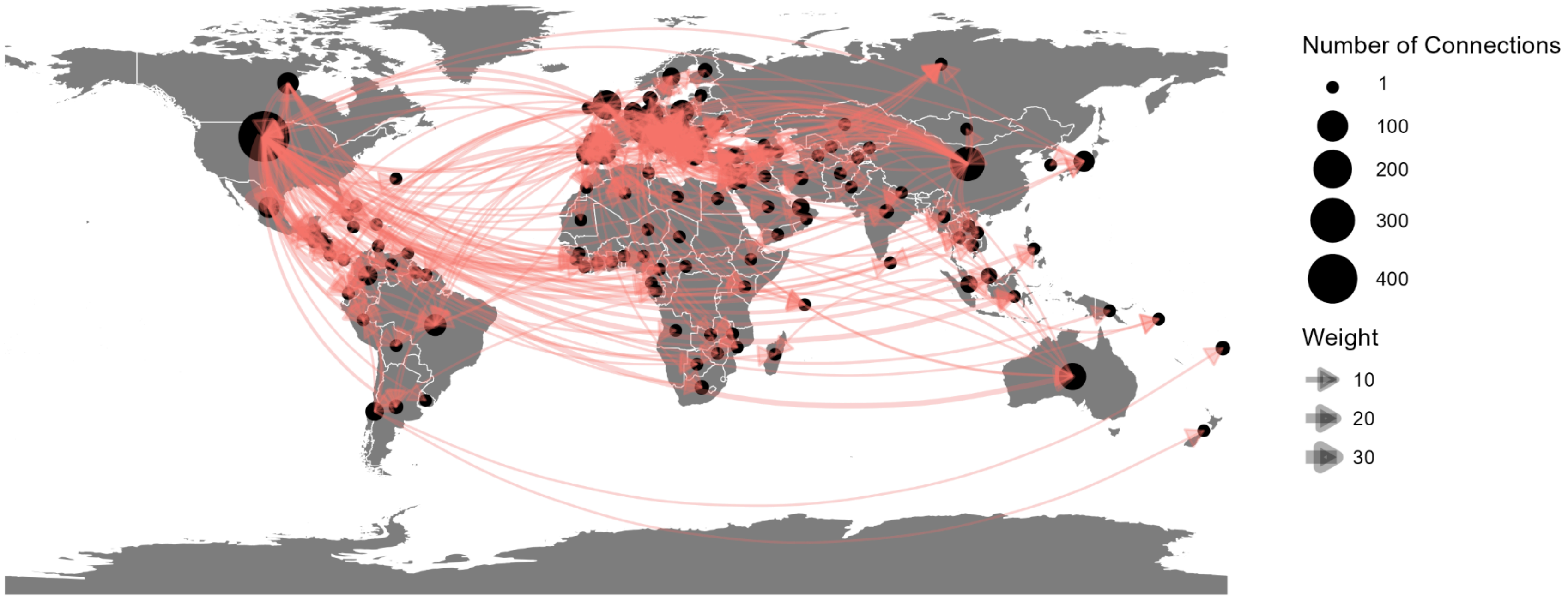

Figure S8. Map of global authorship interactions in genomic studies with international collaborations and sampling locality data (n studies = 303; n genomic resources = 1627). Arrow weights reflect the number of cases where first authors are affiliated with an institution in one country, but published on a study of a species in another country. Circle size represents the total number of connections per country, defined as the sum of first authors that published internationally combined with the number of international authors that published in that country. This figure does not include arrows for entirely domestic (within country) collaborations.

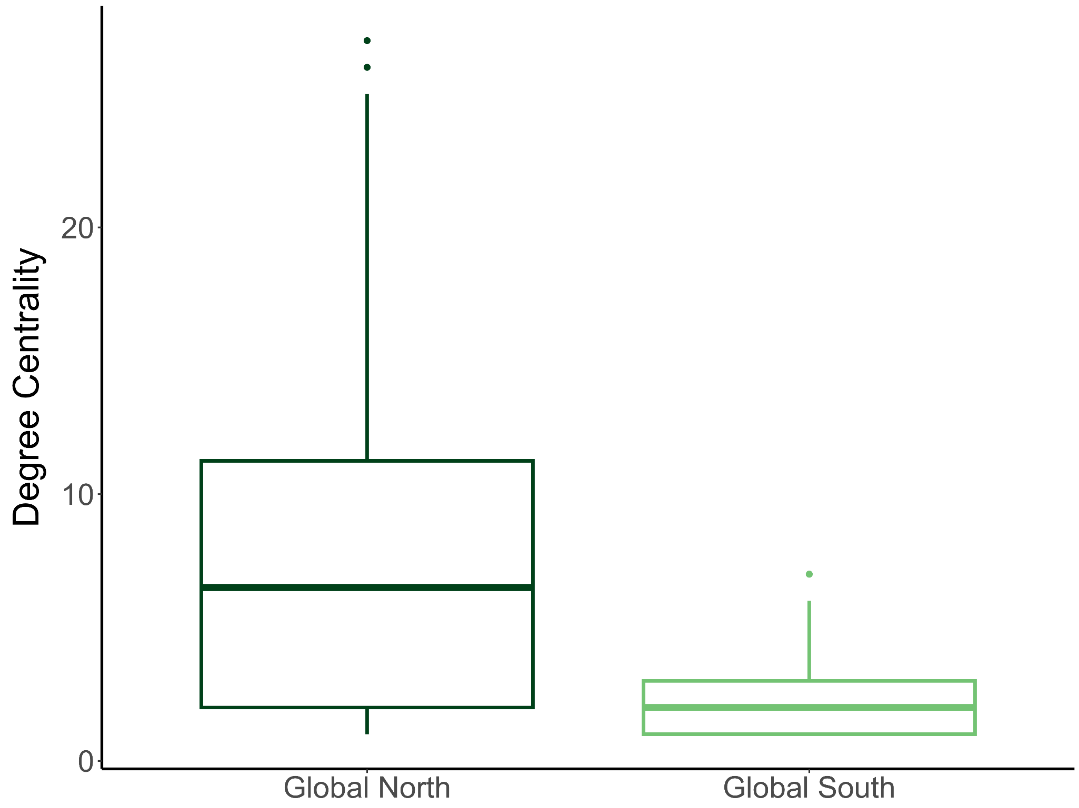

Figure S9. Degree of centrality among papers led by first authors affiliated with institutions in the Global North vs. Global South. Degree of centrality represents the number of international connections, as defined by a paper that includes sampling of a species in a country other than the one in which the first author's affiliated institution is located. A higher degree centrality indicates a greater number of studies including sampling in other countries. This corresponds to the number of links shown in Figure 5 and Figure S8.

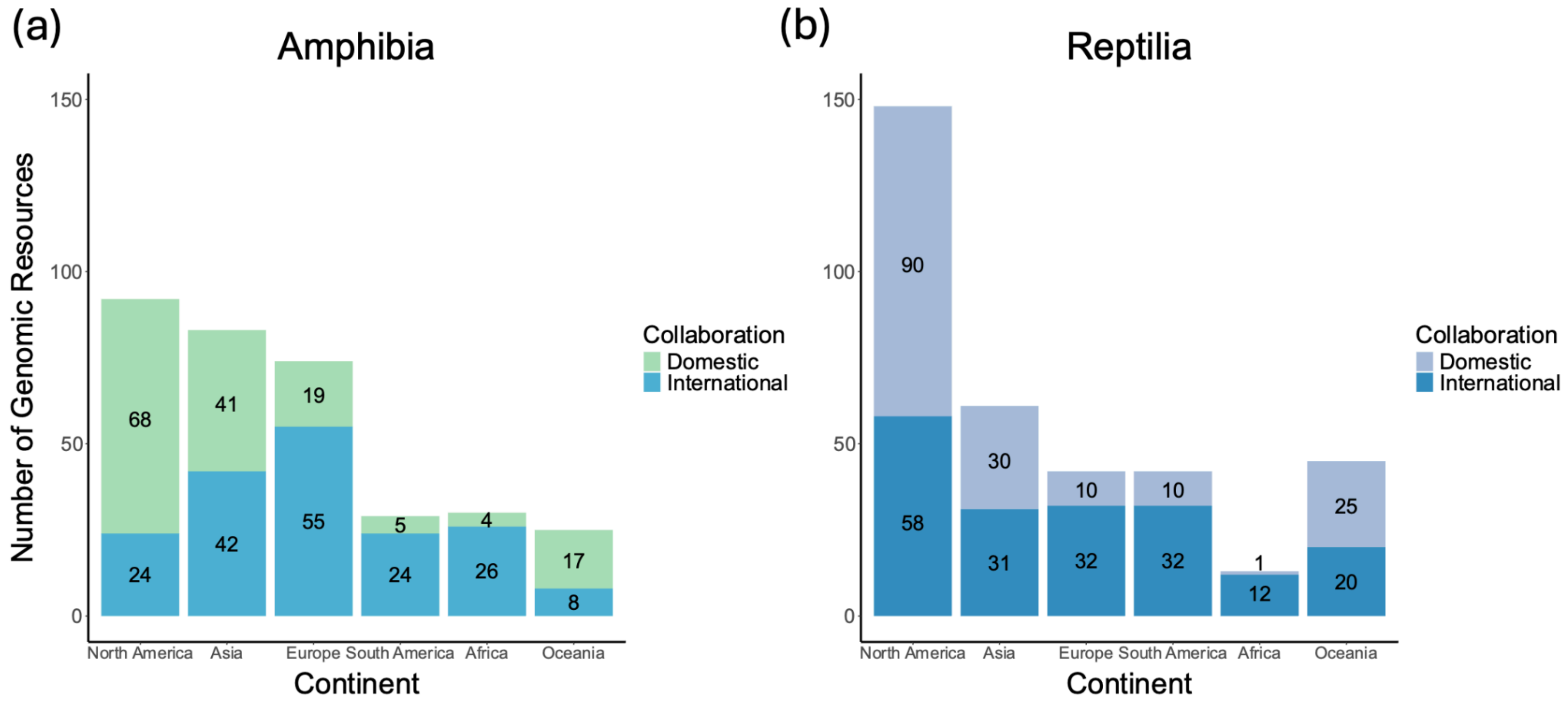

Figure S10. Number of collaborative studies (domestic or international) of wild (a) amphibians (n genomic resources = 333) and (b) reptiles (n genomic resources = 351) with sampling locality data grouped by Continent and collaboration type. This included a total of 606 multi-authored studies, two of which sampled both reptiles and amphibians and were included in both (a) and (b). Bars are ordered according to the overall number of genomic resources; numbers in each bar represent the total number of studies in domestic or international collaboration categories.
